# Fusion in diffusion MRI for improved fibre orientation estimation: An application to the 3T and 7T data of the Human Connectome Project

**DOI:** 10.1016/j.neuroimage.2016.04.014

**Published:** 2016-07-01

**Authors:** Stamatios N. Sotiropoulos, Moisés Hernández-Fernández, An T. Vu, Jesper L. Andersson, Steen Moeller, Essa Yacoub, Christophe Lenglet, Kamil Ugurbil, Timothy E.J. Behrens, Saad Jbabdi

**Affiliations:** a Centre for Functional Magnetic Resonance Imaging of the Brain (FMRIB), University of Oxford, Oxford, UK; b Center for Magnetic Resonance Research, University of Minnesota, Minneapolis, MN, USA; c Wellcome Trust Centre for Neuroimaging, University College London, London, UK

## Abstract

Determining the acquisition parameters in diffusion magnetic resonance imaging (dMRI) is governed by a series of trade-offs. Images of lower resolution have less spatial specificity but higher signal to noise ratio (SNR). At the same time higher angular contrast, important for resolving complex fibre patterns, also yields lower SNR. Considering these trade-offs, the Human Connectome Project (HCP) acquires high quality dMRI data for the same subjects at different field strengths (3T and 7T), which are publically released. Due to differences in the signal behavior and in the underlying scanner hardware, the HCP 3T and 7T data have complementary features in k- and q-space. The 3T dMRI has higher angular contrast and resolution, while the 7T dMRI has higher spatial resolution. Given the availability of these datasets, we explore the idea of fusing them together with the aim of combining their benefits. We extend a previously proposed data-fusion framework and apply it to integrate both datasets from the same subject into a single joint analysis. We use a generative model for performing parametric spherical deconvolution and estimate fibre orientations by simultaneously using data acquired under different protocols. We illustrate unique features from each dataset and how they are retained after fusion. We further show that this allows us to complement benefits and improve brain connectivity analysis compared to analyzing each of the datasets individually.

## Introduction

Determining the optimal acquisition protocol in diffusion magnetic resonance imaging (dMRI) is governed by a series of trade-offs. An increase in the spatial resolution of the acquisition yields lower signal to noise ratio (SNR). An increase in the angular contrast of the images (i.e. higher b value) also reduces SNR. Benefits from improving all these features have been demonstrated. For instance, high spatial resolution reduces partial volume effects and allows exquisite tissue details to be revealed, as has been shown from postmortem acquisitions (Roebroeck et al., 2008, Miller et al., 2011, Leuze et al., 2014), specialised sequences (Heidemann et al., 2012) or in-vivo acquisitions using bespoke scanners (McNab et al., 2013, Sotiropoulos et al., 2013c). High SNR and/or angular contrast are beneficial for accurate and precise estimation of tissue microstructure properties from the dMRI signal, e.g. (Tournier et al., 2004, Behrens et al., 2007, Descoteaux et al., 2009, Sotiropoulos et al., 2012, Zhang et al., 2012). In this paper, we explore whether these trade-offs can be tackled by combining high spatial resolution data with data of higher angular resolution and contrast.

The WU-Minn Human Connectome Project (HCP) (Ugurbil et al., 2013, Van Essen et al., 2013b) has implemented acquisition protocols that improve on both spatial resolution and angular contrast compared to conventional dMRI, while confronting with the limitations imposed by scanning hundreds of subjects. High quality data are publically released at regular intervals. An optimised Connectome Skyra 3T scanner (Siemens, Erlangen, Germany) is being used to acquire data in 1200 subjects with a relatively high spatial resolution (1.25 mm isotropic), multiple angular contrasts (*b*=1000, *b*=2000 and *b*=3000 s/mm^2^) and high SNR (multiple averages and dense sampling in q-space, giving 570 volumes) (Sotiropoulos et al., 2013c). The HCP consortium has also developed acquisition protocols at higher magnetic field strength. A subset of 200 subjects from the population scanned at 3T is also scanned at 7T (Vu et al., 2015) using a 7T MAGNETOM scanner (Siemens, Erlangen, Germany).

The two scanners are equipped with different gradient coils. The maximum gradient strength is 100 mT/m on the 3T WU-Minn Connectome scanner versus 70 mT/m on the 7T MAGNETOM. Due to hardware differences, as well as the different behavior of the MRI signal at different field strengths (e.g. T_2_ relaxation is shorter at 7T, but the baseline SNR is higher (Ugurbil et al., 2013)) the dMRI protocols were designed under different constraints. The 7T data have higher nominal spatial resolution (1.05 mm isotropic), but the 3T have higher angular contrast and angular resolution. Therefore, the 3T and 7T data can provide different views of the same underlying tissue structure. Here, we illustrate some unique and complementary features that the 3T and 7T HCP datasets possess, when analyzed independently. We then propose a data fusion approach for jointly analyzing such dMRI datasets that have been acquired on the same subject with different protocols and different scanners.

We base our approach on an extension of a data fusion generative model that we previously introduced (Sotiropoulos et al., 2013b). This model, called RubiX (Resolutions Unified for Bayesian Inference of X-ings) allows the joint analysis of dMRI data acquired with different spatial resolutions, but using the same b-value, for estimating fibre orientations. We previously showed that, when combining data acquired from the same 3T scanner at different spatial resolutions, the RubiX approach yields more accurate estimates of fibre orientation at the highest of the available resolutions, compared to estimates obtained from data at a single spatial resolution and matched for scanning time. In this paper, we extend the RubiX model to jointly analyse datasets with different spatial and angular samplings, as well as different b-values. We explore the benefits of this model to the publically available HCP data acquired using a 3T and 7T scanner. We show that the analysis of the HCP data can benefit from such a fusion approach that takes advantage of complementarity and retains desired features from both datasets in the estimated orientations.

## Theory

We present an approach for estimating the fibre orientation density functions (fODFs) by simultaneously analysing two datasets of different spatial resolutions and angular contrasts. We subsequently apply this method to jointly analyse 3T and 7T HCP datasets (Table 1) from the same subject. A generative model is used to perform neighborhood-wise parametric spherical deconvolution and allow data fusion. The model has a component that represents the voxel-wise signal at the highest of the available resolutions (i.e. 7T data in this instance) using a convolution integral of the fODF. A different component of the model represents the signal at the lower resolution (i.e. 3T data) using a spatial partial-volume combination of signals from the higher resolution. The two model components allow the two datasets to be combined and are presented in detail in the following sections. They extend our previous work (Sotiropoulos et al., 2013b) in that they allow multiple b shells to be considered and data with different b values and spatial resolutions to be combined.

**Table 1:** Summary of main features of the HCP 3T and 7T dMRI protocols.

	HCP 3T	HCP 7T
Spatial resolution	(1.25 mm)^3^ LR/RL phase encoding (PE)	(1.05 mm)^3^ AP/PA phase encoding (PE)
Acceleration	Multiband = 3 Partial Fourier = 6/8	Multiband = 2, GRAPPA = 3 Partial Fourier = 6/8
Total echo train length	84.24 msec	41 msec
Gradient strength (max)	100 mT/m	70 mT/m
*b* values (s/mm^2^)	1000, 2000, 3000	1000, 2000
Q-space directions	270 × 2 (i.e. each sampled twice with different PEs)	130 × 2 (i.e. each sampled twice with different PEs)

### Voxel-wise spherical deconvolution

Several methods have been proposed for inferring the fODF from diffusion MRI. The underlying idea is that the signal measured in anisotropic white matter *S*^*AN*^ can be considered as the spherical convolution of the fODF *F* and an impulse response function *R*, i.e. *S*^*AN*^(*θ*, *φ*) = *F*(*θ*, *φ*) ⊗ *R*(*θ*, *φ*), where *θ* and *φ* are the inclination and azimuth angles on the sphere. Estimating *F* is a deconvolution operation (Behrens et al., 2003, Tournier et al., 2004, Alexander, 2005, Anderson, 2005, Dell'Acqua et al., 2007, Kaden et al., 2007, Sotiropoulos et al., 2012, Zhang et al., 2012). Deconvolution methods differ in the way they represent *F* (parametrically or non-parametrically, e.g. using some basis functions) and treat *R* (measure or explicitly model it).

We represent the signal attenuation within a voxel due to the *k*^*th*^ diffusion-sensitising gradient (*k* = *1*:*K*) as a weighted sum of the attenuation from a partial volume component (*E*^*PV*^) and from an anisotropic component (*E*^*AN*^):

(1) $$S\left(\theta_{k},\varphi_{k}\right)/S_{0}=S_{k}/S_{0}=\left(1-f_{\text{AN}}\right)E_{k}^{\mathrm{PV}}+f_{\text{AN}}{\;E}_{k}^{\text{AN}}$$

with *S*_0_ being the signal intensity without any diffusion-weighting and 0 ≤ *f*_*AN*_ ≤ 1 the fraction of the signal explained by the anisotropic component.

Partial volume has been represented before using an isotropic diffusion compartment of a single diffusivity (for instance (Behrens et al., 2007, Dell'Acqua et al., 2007, Kaden et al., 2007)) or ignored (Tournier et al., 2007). We use a distribution of isotropic diffusion compartments, as in (Jbabdi et al., 2012). This is a phenomenological representation that adds only a single parameter, yet allows the model to capture non-monoexponential signal decay with b value and represent data acquired with multiple *b* values (Lasic et al., 2014). Assuming a Gamma distribution of diffusivities with shape and scale parameters *α* and 1/*β* gives:

(2) $$E_{k}^{\mathrm{PV}}={\left(\frac{\beta }{\beta +b_{k}}\right)}^{\alpha }$$

with *b*_*k*_ the *b* value of the *k*^th^ diffusion-sensitising gradient and the Gamma parameters relating to the mean diffusivity *d*_*m*_ and variance of the distribution *d*_*std*_^2^ as *β* = *d*_*m*_/*d*_*std*_^2^ and *α* = *d*_*m*_*β*.

The anisotropic component is described using a spherical convolution integral of the fODF *F*:

(3) $$E^{\text{AN}}=\int_{0}^{2\pi }\int_{0}^{\pi }F\left(\theta ,\varphi \right)R\left(\theta ,\varphi \right)\sin\theta \mathrm{d\theta d\varphi}.$$

We represent the convolution kernel *R* as the signal attenuation from an axially symmetric anisotropic tensor *D* = (*λ*_1_ - *λ*_2_)***v***^***T***^***v*** + *λ*_2_**I**_3_, with eigenvalues *λ*_1_ ≫ *λ*_2_ and *λ*_2_ = *λ*_3_, principal orientation ***v*** = [sin ⁡ θ cos ⁡ φ sin ⁡ θ sin ⁡ φ cos ⁡ θ] and **I**_3_the 3 × 3 unit matrix (Anderson, 2005). Re-parameterising using the mean of the eigenvalues *λ*_*m*_ and their ratio *λ*_*R*_ = *λ*_2_/*λ*_1_ gives:

(4) $$R_{k}\left(\theta ,\varphi \right)=\exp\left[-b_{k}\frac{3\lambda_{m}}{2\lambda_{R}+1}\left(\left(1-\lambda_{R}\right){\left(g_{k}v^{T}\right)}^{2}+\lambda_{R}\right)\right]$$

with ***g***_*k*_ a unit row vector representing the orientation of the *k*^*th*^ diffusion-sensitising gradient.

Finally, the fODF is represented using a sum of *N* spherical delta functions, each oriented along an orientation ***v***_*n*_, allowing direct deconvolution against the fODF maxima (Behrens et al., 2007). The convolution integral then reduces to a summation of *N* fibre components ($f_{\mathrm{AN}}=\sum_{n=1}^{N}f_{n}\leq 1)$. Assuming that these *N* fibre components are characterised by the same convolution kernel *R*, the final model for the signal becomes:

$$S_{k}=S_{0}\left[\left(1-\sum_{n=1}^{N}f_{n}\right){\left(\frac{d_{m}}{d_{m}+b_{k}\;d_{std}^{2}}\right)}^{d_{m}^{2}/d_{std}^{2}}\right)$$

(5) $$\left(+\sum_{n=1}^{N}f_{n}e\mathrm{xp}\left(-b_{k}\frac{3\lambda_{m}}{2\lambda_{R}+1}\left(\left(1-\lambda_{R}\right){\left(g_{k}v_{n}^{T}\right)}^{2}+\lambda_{R}\right)\right)\right]$$

Using the above model and a set of voxel-wise (multi-shell) measurements, we can estimate the unknown parameters, with the vectors ***v***_*n*_ being the main parameters of interest that represent the fODF maxima.

### Neighborhood-wise deconvolution

The above approach considers the measurements and model parameters individually at every voxel, as most spherical deconvolution methods do. Another approach has been introduced in Sotiropoulos et al., (2013b), where multiple voxels are simultaneously considered. In the presence of datasets acquired at different spatial resolutions (and therefore SNRs), the low-resolution (LR) and high-resolution (HR) data are fused in a Bayesian hierarchical model to allow deconvolution of the fODFs defined at the highest of the available resolutions (HR). This approach has the potential to provide more accurate and precise estimation at HR compared to voxelwise estimation, as it can take advantage of complementarity in the fused datasets.

We use this framework to fuse the 7T (HR) and 3T (LR) HCP datasets acquired from the same subject (datasets having *K* and *L* q-space samples respectively). The RubiX generative model comprises of a local voxel-wise convolution representation (Eq. (5)) and a neighborhood-wise spatial partial volume representation (see Eq. (6) below). The former allows predictions to be made for the HR dataset using parameters defined at the HR grid. The local and the spatial representations together allow predictions to be made for the LR dataset using the same set of HR model parameters.

Assuming that the HR and LR voxel grids have been aligned into the same physical space and that an LR voxel intersects *P* HR voxels, each with fraction *a*_p_ ($\sum_{p=1}^{P}a_{p}=1$), then:

(6) $$\frac{S_{l}^{\mathrm{LR}}}{S_{0}^{\mathrm{LR}}}=\frac{\sum_{p=1}^{P}a_{p}S_{l,p}^{\mathrm{HR}}}{\sum_{p=1}^{P}a_{p}S_{0,p}^{\mathrm{HR}}}$$

where *S*_*l*_^*LR*^ and *S*_*l*,*p*_^*HR*^ are the signals from the *l*^*th*^ (*l* = *1*:*L*) diffusion-sensitising gradient for the LR voxel and the *p*^*th*^ HR voxel it intersects, respectively. Notice that the *L* and *K* diffusion-sensitising gradients can be different between the two datasets. Eq. (6) combined with Eq. (5) can provide signal predictions for the *l* = *1*:*L* measurement points of the LR dataset (for every HR voxel *p*, *S*_0,*p*_^*HR*^ is the model parameter *S*_0_ in Eq. (5) and Eq. (5) can be evaluated at the *l*^th^ measurement point (*b*_*l*_, ***g***_*l*_) to provide a prediction *S*_*l*,*p*_^*HR*^). Also, Eq. (5) alone can provide predictions *S*_*k*_^*HR*^ for the *k* = *1*:*K* measurement points (*b*_*k*_, ***g***_*k*_) of the HR dataset. Therefore, the two equations combined can explain both datasets acquired with gradients that can be different in magnitude and direction. This allows different spatial resolution/angular contrast/SNR trade-offs to be combined.

The spatial model used in this study (Eq. 6) is slightly different from the one used in (Sotiropoulos et al., 2013b), which instead uses a weighted summation of the attenuations: *S*_*l*_^*LR*^/*S*_0_^*LR*^ = ∑_*p*=1_^*P*^*a*_*p*_*S*_*l*,*p*_^*HR*^/*S*_0,*p*_^*HR*^. Both spatial representations assume that T1 and T2 vary smoothly in the neighborhood that is considered. However, Eq. (6) is less sensitive to averaging signal from different tissue types and provides more accurate estimates particularly at tissue boundaries (see simulations presented in the Supplementary Material, Figure S2).

The signal for the HR voxels is represented using the local convolution model described before. Thus, Eqs. (5), (6) provide a generative model for predicting both LR and HR datasets given parameters at the HR only.

### Inference

We use Bayesian inference and a Metropolis MCMC algorithm to invert the above forward model and estimate the posterior distribution *P*(***Ω*** |*** Y***) of the set of unknown model parameters ***Ω*** given a set of measurements ***Y***. Using Bayes' theorem, *P*(***Ω*** |*** Y***) ∝ *P*(***Y*** |** Ω**) *P*(**Ω**), i.e. the posterior distribution of the model parameters is proportional to the likelihood of the measurements and the prior distribution of the parameters.

To define the likelihood function, we assume zero-mean, additive Rician noise with precision τ = 1/*σ*^2^ (*σ*^2^ the noise variance). Then, for each measurement *Y*_*k*_ of the HR dataset, the likelihood will follow a Rician distribution centered around the respective model prediction *S*_*k*_, i.e. *P*(*Y*_*k*_ |*** Ω*** , *τ*) ~ *Rice*(*S*_*k*_, *τ*). Assuming the measurements are independent from each other, the likelihood for a set of *k* = *1*:*K* (similarly for *l* = *1*:*L*) measurements *Y*_*k*_ is given by the product of the individual likelihoods:

(7) $$P\left(\left(Y^{\mathrm{HR}}\right|\Omega ,\tau \right)=\prod_{k=1}^{K}{\mathrm{\tau Y}}_{k}e\mathrm{xp}\left(-\tau \left(Y_{k}^{2}+S_{k}^{2}\right)/2\right)I_{0}\left({\mathrm{\tau Y}}_{k}S_{k}\right)$$

with *I*_*0*_*()* the 0th order modified Bessel function of the first kind and *S*_*k*_ the predictions using the local model (Eq. 5). When considering both HR and LR datasets (again assuming independence), the joint likelihood of both datasets for a LR voxel and the group of HR voxels intersecting it is given by:

(8) $$P\left(Y^{\mathrm{LR}},\left\{Y_{p}^{\mathrm{HR}}\right\}\;\right|\;\Omega^{\mathrm{All}},\tau^{\mathrm{LR}},\left\{\tau_{p}^{\mathrm{HR}}\right\})=P\left(Y^{\mathrm{LR}}\;\right|\Omega^{\mathrm{All}},\tau^{\mathrm{LR}})\prod_{p=1}^{P}P\left(Y_{p}^{\mathrm{HR}}|\Omega_{p}^{\mathrm{HR}},\tau_{p}^{\mathrm{HR}}\right)$$

where we assume a Rician noise distribution for both datasets with different precision (noise) levels *τ*, ***Ω***^***All***^ = {{***Ω***_***p***_^***HR***^}_***p***=1:***P***_, ***S***_0_^***LR***^} and ***Ω***_***p***_^***HR***^ the set of unknown model parameters for the *p*^th^ HR voxel, as defined in Eq. (5). I.e. for a single HR voxel the unknown model parameters will be ***Ω***^*HR*^ = {*S*_0_^*HR*^, *d*_*m*_, *d*_*std*_, *λ*_*R*_, {*f*_*n*_, ***v***_*n*_}_*n*=1_^*N*^}. The likelihood for the LR data *P*(***Y***^***LR***^ |*** Ω***^***All***^ , ***τ***^***LR***^) will be given as above (Eq. 7) with the product evaluated over the *l* = *1*:*L* measurements *Y*_*l*_ and the predictions *S*_*l*_ obtained from the spatial and local model (Eqs. (5), (6)).

The prior distributions need to be also defined. A-priori (*S*_0_^*HR*^) ∼ *U*(0, ∞), *P*(*S*_0_^*LR*^) ∼ *U*(0, ∞), *P*(*τ*^*HR*^) ∼ *U*(0, ∞) and *P*(*τ*^*LR*^) ∼ *U*(0, ∞) allowing positive values and *P*(***v***) ∝ sin*θ*, such that ***v*** is uniform on the sphere. The principal volume fraction *f*_1_ is also uniformly distributed on [0, 1], while an automatic relevance determination (ARD) prior that encourages sparsity in the fODF (Wipf and Nagarajan, 2008) is used for the other volume fractions, *P*(*f*_*n*_) ∝ 1/*f*_*n*_^*w*^, *n* ≥ 2. This allows on-the-fly determination of the fODF complexity, with multi-modal fibre patterns being a-priori penalised(Behrens et al., 2007). The hyper-parameter *w* controls the severity of the penalty with *w* < 1 relaxing and *w* > 1 increasing the penalty compared to the *w* = 1 case.

**Fig. 1:**
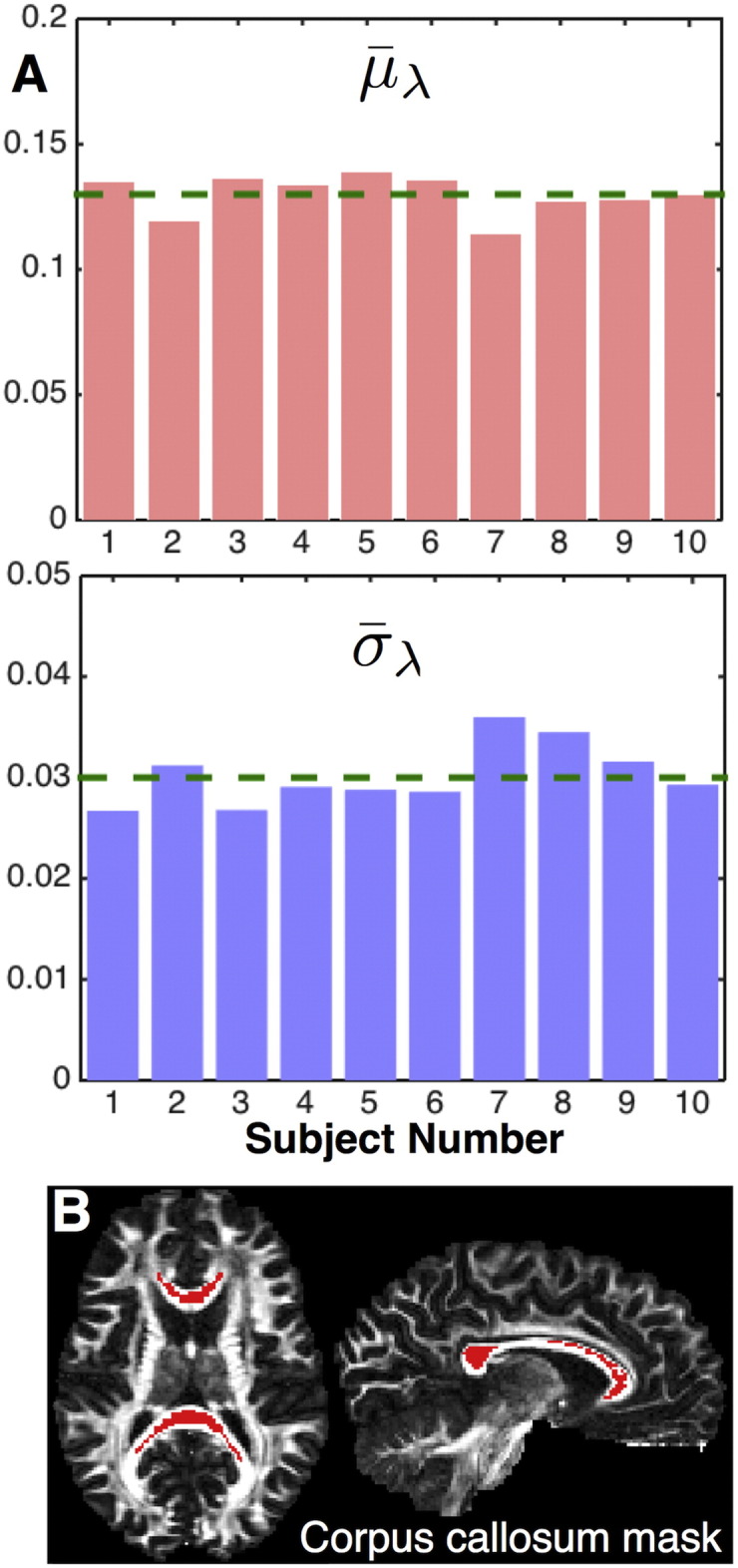
A) Mean and standard deviation of the anisotropy ratio (λ_R_ = λ_radial_/λ_axial_) across ten different HCP subjects. The values for each subject were obtained using the DTI eigenvalues in a region depicting the most anisotropic voxels in the corpus callosum (red mask in B). The green dashed lines correspond to the average values used for the hyperparameters of the λ_R_ prior distribution (μ_λ_ = 0.13, σ_λ_ = 0.03). B) Axial and sagittal views of the mask depicting the most anisotropic voxels of the corpus callosum. The midbody of the corpus callosum was identified in the Johns Hopkins University white matter atlas (Wakana et al., 2007), available in FSL. The mask was eroded once and voxels with the top 50% FA values within this mask were retained. The mask is shown in red superimposed on an FA map.

The priors used for the diffusivity parameters are informative to make the partial volume and the white matter compartments with varying anisotropy identifiable and avoid “competition” between the two. For the same reason, we set *d*_*m*_ = *λ*_*m*_ (i.e. both compartments share the same mean diffusivity) and allow large differences in mean diffusivities to be captured by *d*_*std*_. An ARD prior is used for the *d*_*std*_ parameter (i.e. *P*(*d*_*std*_) ∝ 1/*d*_*std*_) to a-priori encourage a single diffusion coefficient *d*_*m*_ within a voxel. A Gamma prior is used for the diffusivity *d*_*m*_ with a mean around 10^−3^ mm^2^/s (shape = 3, scale = 0.25 × 10^−3^) to encourage diffusivities observed in the brain parenchyma (Pierpaoli and Basser, 1996). Finally, a Gaussian prior is used for the parameter *P*(*λ*_*R*_) ~ *N*(*μ*_*λ*_, *σ*_*λ*_^2^). Given that this prior is on the anisotropy of the convolution kernel *R*, we learn its parameters from the data. We assume that the corpus callosum is probably the most coherently organised white matter region and we use this region to learn features about the “single-fibre” response. This is similar to the approach used by Tournier et al. (2004), except that instead of measuring the response function directly form the corpus callosum and fixing it in the model, we learn prior hyper-parameters for the response function. We use an FA threshold to obtain the most anisotropic voxels within the corpus callosum (defined by an eroded MNI mask) and find the mean and standard deviation of the parameter *λ*_*R*_ across the voxels using the diffusion tensor eigenvalues (*λ*_*R*_ = (*λ*_2_ + *λ*_3_)/2*λ*_1_). These values across 10 HCP subjects are shown in Fig. 1, along with an example of the corpus callosum mask.

The RubiX model uses some informative priors to further combine the two datasets. Briefly, the prior on the orientations is a sum of Watson distributions. Crucially, the modes and variances of these priors are common to a neighborhood of *P* voxels and are all estimated on-the-fly from the data as hyper-parameters. They therefore impose spatial constraints on the HR estimates, where the constraints themselves are estimated from the data. An intuitive description for this prior is that orientation information from the LR data (that have more angular contrast/coverage) is learned in the hyperparameters, which then constraints the solution space at the HR grid, see (Sotiropoulos et al. 2013b) for the full details.

Having defined both the likelihood function and the prior distributions the posterior distribution of the model parameters given the measurements is defined up to a proportionality constant. We use Markov-Chain Monte-Carlo (MCMC) to estimate this posterior, as shown before (Behrens et al., 2003, Behrens et al., 2007).

## Methods

### Data acquisition

Scans were performed in a 3T Siemens Connectome Skyra and a 7T Siemens MAGNETOM, equipped with a 100 mT/m and a 70 mT/m gradient set, respectively, and 32-channel receive coils (Ugurbil et al., 2013). Details of the 3T and 7T dMRI acquisition protocols are given in Sotiropoulos et al. (2013c) and Vu et al. (2015), with main scan parameters summarised in Table 1. Briefly, the HCP dMRI scans utilise the Stejskal-Tanner pulsed gradient (i.e. monopolar) scheme (Stejskal and Tanner, 1965), within a single-shot 2D spin-echo multiband EPI acquisition. A SENSE1 magnitude reconstruction is used so that datasets have Rician noise (Sotiropoulos et al., 2013d). For the 3T, nominal spatial resolution is 1.25 mm isotropic (matrix size PE × Readout = 144 × 168 with left–right (LR) phase encoding (PE) and 6/8 PE partial Fourier), with 111 slices acquired in interleaved slice order to cover the entire brain using a Multiband (MB) factor of 3 for slice acceleration and no acceleration along the PE direction. A total of 108 echoes are collected, with echo spacing of 0.78 ms and readout bandwidth 1490 Hz/pixel, resulting in a total echo train length (ETL) of 84.24 ms. Sampling in q-space includes 3 shells at *b* = 1000, 2000 and 3000 s/mm^2^ (diffusion times are Δ = 43.1 ms and δ = 10.6 ms). TE and TR are matched across shells (TE = 89 ms, TR = 5.5 s). For each shell, 190 data points are obtained, corresponding to 90 isotropic diffusion-sensitised directions and 5 *b* = 0′s acquired once per phase encoding (PE) direction (i.e. LR and RL pairs). Total scanning time for this protocol is ~ 55 min.

For the 7T, nominal spatial resolution is 1.05 mm isotropic (matrix size PE × Readout = 200 × 200 with 6/8 PE partial Fourier), with 132 slices acquired in interleaved slice order to cover the entire brain and phase encoding applied along the anterior–posterior (AP/PA) direction, using MB = 2 and PE acceleration (GRAPPA) of 3. A total of 50 echoes are collected, with echo spacing of 0.82 ms and readout bandwidth 1388 Hz/pixel, resulting in a total echo train length (ETL) of 41 ms. Sampling in q-space includes 2 shells at *b* = 1000, 2000 s/mm^2^ (diffusion times are Δ = 34 ms and δ = 14.3 ms). TE and TR are matched across shells (TE = 71 ms, TR = 7 s). For each shell, 142 data points are obtained, corresponding to 65 diffusion-sensitised directions and 6 *b* = 0′s acquired once per phase encoding direction (i.e. AP and PA pairs). Total scanning time for this protocol is ~ 40 min.

**Fig. 2:**
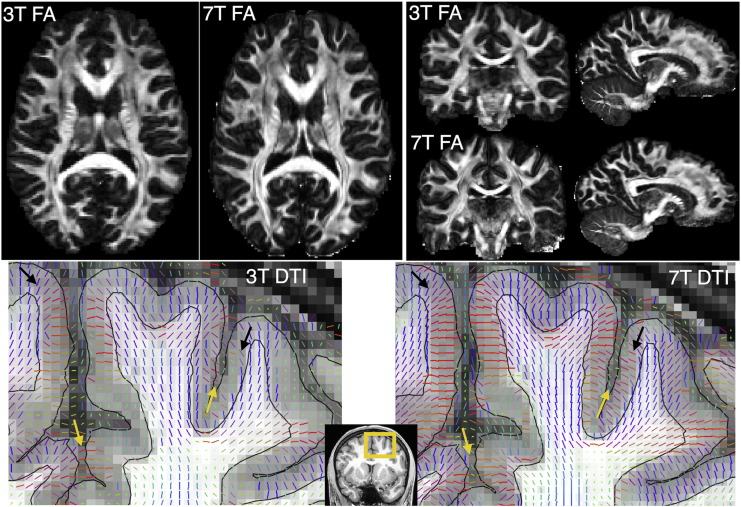
DTI maps for 3T and 7T data of the same subject (HCP Subject ID: 158035). Top row: Fractional anisotropy (FA) maps (axial, coronal and sagittal views). Notice the effect of B1 inhomogeneities for the 7T that lead to poor SNR and noisy FA estimates at the inferior temporal regions (evident in the coronal views). These artifacts are present for the 7T data, but efforts have been taken to minimise them (Vu et al., 2015). Bottom row: DTI principal fibre orientations (coronal zoomed view of the area delineated by the yellow box). The orientations are RGB color-coded (Red: Left–Right, Green: Anterior–Posterior, Blue: Superior–Inferior) and superimposed on the structural T1w image. The pial surface and the WM/GM boundary surface are also shown.

The 3T data contain more angular information (due to both the higher gradient strength and the longer scanning time available) and are expected to provide better sensitivity to detecting crossing fibres and resolving complex fODFs. On the other hand, the 7T data have higher spatial resolution. Despite the seemingly small difference in voxel size (which is still a ~ 40% decrease in nominal voxel volume), the 7T data are also closer to their nominal resolution (Vu et al., 2015). The utilisation of in-plane GRAPPA produces a shorter echo train length, which leads to smaller PSF blurring along the phase-encoding direction. This results in a true resolution gain of ~ 44% in voxel volume (Vu et al., 2015). Fig. 2 shows qualitatively the difference in crispness in the FA maps (top row). DTI fibre orientations are also shown in addition to the pial and WM/GM boundary surfaces. Notice that the fibre spreading pattern to the cortex as well as orientation information within the cortical ribbon (black arrows) are better depicted in the 7T data. The 7T also support better separation between neighbouring gyri at certain areas (yellow arrows).

### Processing and analysis

Both datasets were corrected for distortions using the HCP dMRI pipelines (Glasser et al., 2013, Sotiropoulos et al., 2013c). An approach based on Gaussian processes was used to combine the PE pairs and correct for susceptibility (Andersson et al., 2003), eddy-current and head motion induced distortions (Andersson and Sotiropoulos, 2015, Andersson and Sotiropoulos, 2016). The datasets were also corrected for gradient non-linearities and were both aligned to the 3T T1 structural space using spline interpolation. The 3T dMRI dataset was registered to the T1 using a rigid body transformation. The 7T dataset was registered using a linear transformation with 9 degrees of freedom, as a rigid body transformation was not enough to account for slight gradient miscalibrations between the two scanners. (In general, scalings in the order of 1–3% along the different axes were found necessary to perfectly register images between the different Siemens scanners, even when considering corrections for gradient non-linearities, as in Glasser et al. (2013)). All registrations were boundary-based (Greve and Fischl, 2009), as these were found superior to volumetric. The directions of the diffusion-sensitising gradients were reoriented using the rotational components of the transformations.

Fibre orientations were estimated using either voxel-wise deconvolution (Eq. 5), applied independently to each of the 3T or 7T datasets (up to *N* = 3 fibre compartments estimated), or the RubiX model applied to both 3T and 7T datasets simultaneously (up to *N* = 3 fibre compartments estimated with an orientation prior with 3 modes). The idea behind neighborhood-wise deconvolution allows in principle arbitrary low and high resolutions to be combined. Due to the dimensionality of the problem and the large datasets to be combined, we developed a parallel implementation to achieve practical execution times. We used the CUDA principles outlined in Hernandez et al. (2013) to run the RubiX MCMC-based inference on Graphics Processing units (GPUs). To allow parallelisation, this implementation assumes an integer volume ratio of LR and HR voxels, implying *a*_*k*_ = 1/*P* (see Eq. 6) and that each HR voxel intersects only one LR voxel; or that *P* HR voxels fit perfectly within one LR. That way, neighborhoods of *P* voxels can be treated independently and inference can be parallelised. Thus, we downsample the LR (3T data) to twice the spatial resolution of the 7T when running RubiX. Broadly, given that the LR data affect more directly the estimation of the orientation prior hyper-parameters in the RubiX framework (see (Sotiropoulos et al., 2013b) for details) and their angular information is what complements the already high spatial resolution of the 7T data, we chose to sacrifice the 3T spatial resolution in order to achieve estimation in realistic time frames. The MCMC was then run with a burn-in period of 5000 iterations, with 1250 extra iterations for sampling. Thinning of the MCMC posteriors and reduction of the autocorrelation of the chains was achieved by retaining only every 25th sample. Computation times for the very high-resolution HCP datasets of a single subject were in the order of 4 h using 6 GPUs (about an order of magnitude longer than performing Bayesian Inference using a single resolution local model without any data fusion).

## Results

In this section, we illustrate examples of desirable features from each of the two datasets that are preserved after data fusion. Such features include the higher sensitivity to fibre complexity of the 3T and the higher spatial resolution of the 7T.

**Fig. 3:**
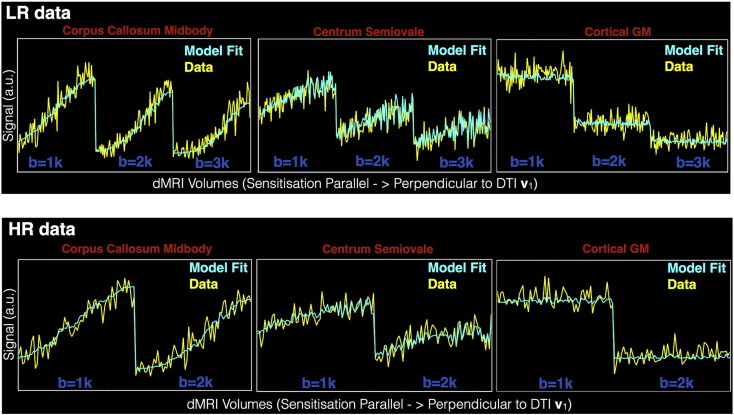
RubiX model predictions of both LR (3T) and HR (7T) datasets for three different locations (in the midbody of the corpus callosum, in the centrum semiovale and in cortical grey matter). For each model parameter, the mode of the estimated posterior distribution was used to obtain model predictions. The volumes have been grouped into b-shells. Within each shell, the volumes have been rearranged according to the dot product between the corresponding gradient direction and the principal DTI eigenvector.

First, we show the ability of the RubiX model to perform reasonable predictions for both datasets given the set of parameters ***Ω***^*All*^. Fig. 3 presents model predictions along with data at different locations in the brain. To obtain the predictions, we used the mode of the estimated posterior distribution of each model parameter. We then used the gradient direction scheme of each acquisition and Eqs. (5), (6) to predict the signal at 3T and 7T. The data points have been grouped into b-shells. Within each shell, they have been reordered according to the angle of the corresponding gradient direction with the principal DTI eigenvector. Thus, the points are shown sorted from parallel to perpendicular to the principal diffusion direction. Despite the differences in the acquisition protocols, the model predicts reasonably well both datasets.

**Fig. 4:**
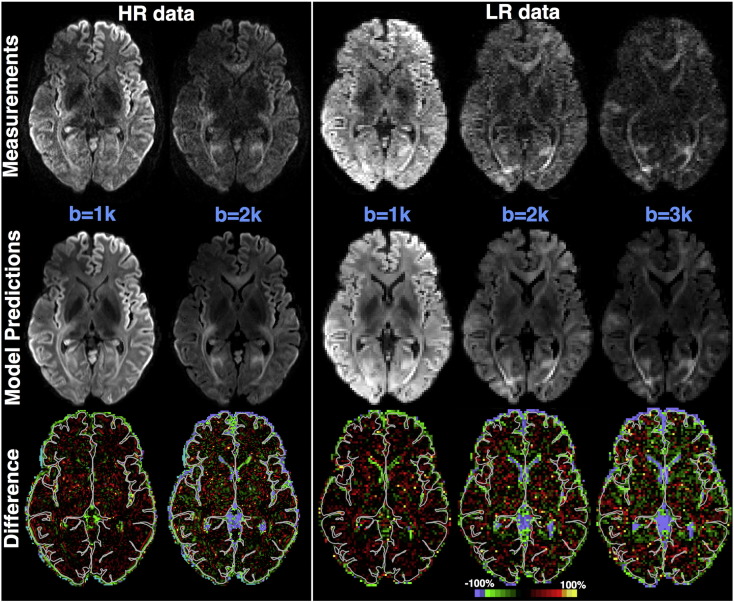
RubiX model predictions of different measurement volumes in the HR (7T) and LR (3T) data. The top row shows the actual measurements (the 3T data have been downsampled, as used in the estimation). The middle row shows the predictions. To obtain model predictions, the mode of the estimated posterior distribution was used for each model parameter. The bottom row shows the difference of the prediction minus the measurement expressed as a percentage of the measurement. The pial surface is shown as a grey outline (HCP Subject ID: 102311).

Alternatively, instead of focusing on all measurements at single locations, Fig. 4 shows predictions for a single measurement volume. For both datasets, the diffusion contrast in the predicted volumes follows the measured ones. The bottom row shows the difference between prediction and measurements, expressed as a % fraction of the measurement. The largest differences can be observed in the CSF-filled areas (either in the ventricles or at the brain periphery outside the pial surface, as indicated by the grey line) and these further increase with b-values. The model predicts a lower CSF signal than measured, with the measurements however reflecting an elevated noise floor. Within the brain tissue, the absence of a consistent bias towards higher or lower signal predictions can be noticed. In regions with larger inhomogeneities (see Supplementary figures S4, S5), the deviations of the model predictions from the data are higher, yet the predictions are reasonable for both datasets and all *b* values.

### Preserving the higher fibre complexity supported by the 3T data

**Fig. 5:**
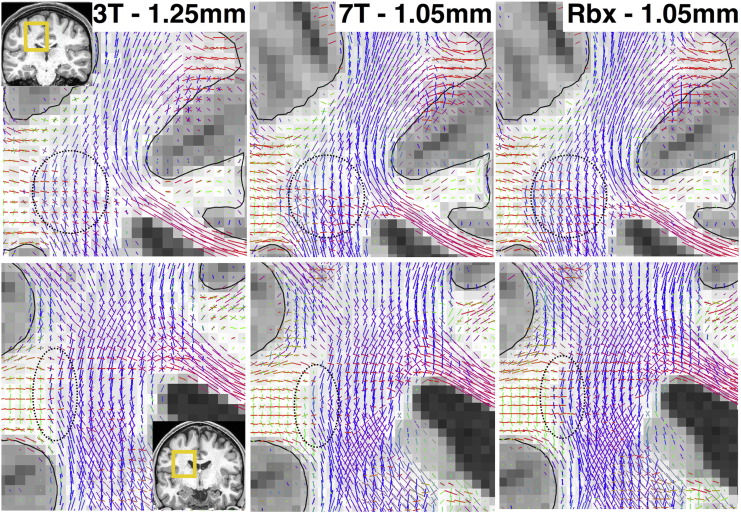
Fibre orientations in the centrum semiovale (coronal views) estimated using (from left to right): Voxel-wise deconvolution on the 3T alone (estimates at nominal spatial resolution (1.25 mm)^3^, Voxel-wise deconvolution on the 7T alone (estimates at nominal spatial resolution (1.05 mm)^3^), RubiX deconvolution on both 3T and 7T (estimates at nominal spatial resolution (1.05 mm)^3^). The two rows correspond to two different subjects. The black contour represents the WM/GM boundary surface. The vectors have been RGB color-coded and their norm is modulated by the respective volume fractions. Only fibres with volume fraction f > 5% are shown.

Fig. 5 shows a qualitative comparison between the peaks of the fODF that have been estimated using three different approaches; voxel-wise deconvolution (i.e. Eq. (5)) using the 3T data alone (estimates at nominal spatial resolution (1.25 mm)^3^), voxel-wise deconvolution using the 7T alone (estimates at nominal spatial resolution (1.05 mm)^3^) and RubiX using both 3T and 7T (estimates at nominal spatial resolution (1.05 mm)^3^). The estimated orientations in the centrum semiovale are shown for two different subjects (top and bottom row). Using the 3T data, which have rich angular information, we can estimate two-way and three-way crossings at the native 3T resolution, as expected. Using the 7T data alone, we can still get reasonable crossing estimates, but the orientations look more spatially incoherent at certain places and some crossings are missing (example areas are depicted within the dotted circles). After data fusion, we estimate very coherent crossings at the resolution of the 7T grid i.e. we can get equally coherent fibre patterns as with the 3T data, but at a higher spatial resolution.

**Fig. 6:**
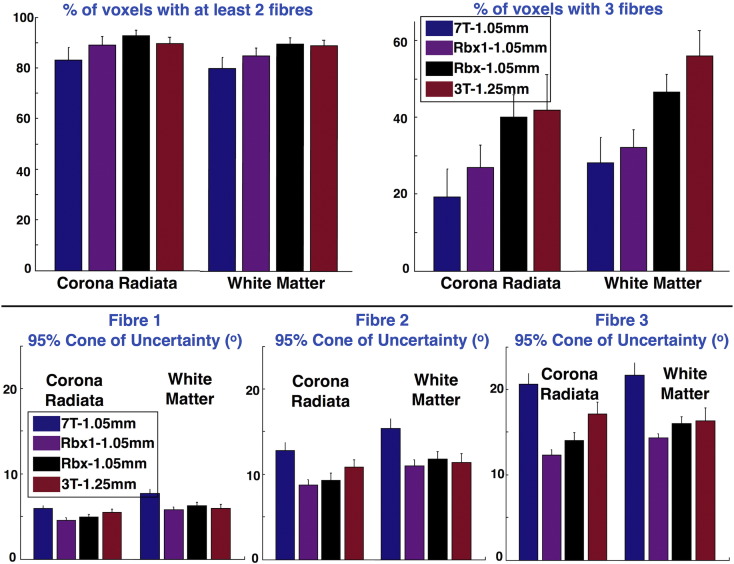
Number of crossing fibres (top row) and uncertainty of estimated orientations (bottom row) obtained using four different methods: voxel-wise deconvolution at 3T (red), voxel-wise deconvolution at 7T (blue), RubiX deconvolution using both 3T and 7T and *w* = 1 for model selection (Rbx1, purple) and RubiX deconvolution using *w* = 0.8 (Rbx, black). Notice the difference in the spatial resolution, on which each method operates and estimates fibre orientations. Two ROIs have been considered, one in the corona radiata (obtained from the Johns Hopkins University white matter atlas (Wakana et al., 2007)) and one covering the white matter. The bars correspond to the mean and standard deviation across 10 subjects. For each subject and each ROI, the mean values were obtained. Only fibre orientations with f > 5% were considered. The percentages at the top row are expressed as fractions of the total ROI volume. The uncertainties at the bottom correspond to the 95% cone of uncertainty (Jones, 2003).

The data fusion further permits an increase in the precision of orientation estimates and/or an increase in the sensitivity in detecting fibre crossings at high resolution. To quantify these gains, we counted the amount of crossing fibre patterns that have been detected in white matter using the three approaches. We also computed the 95% cone of uncertainty (Jones, 2003) for each estimated orientation. Fig. 6 illustrates the results across ten subjects and two ROIs (corona radiata and whole white matter). Two versions of RubiX are included along with the 3T and 7T results that correspond to different values for the model selection sensitivity parameter (*w* = 1: standard ARD prior and *w* = 0.8: relaxed ARD prior). In all cases, RubiX increases the sensitivity in detecting crossings at HR (particularly 3-way, top row) and the precision of the HR estimates (bottom row) compared to results obtained from the 7T data alone. By relaxing the ARD prior (i.e. use *w* < 1), we can allow more crossing fibres to be detected at the expense of estimation precision. We have chosen *w* = *0.8* as a middle-ground solution, which allows RubiX estimates to be as precise as the 3T estimates, have a sensitivity in detecting crossings close to the 3T, while being at higher resolution than the 3T.

### Preserving the fibre spreading pattern to the cortex supported by the 7T data

**Fig. 7:**
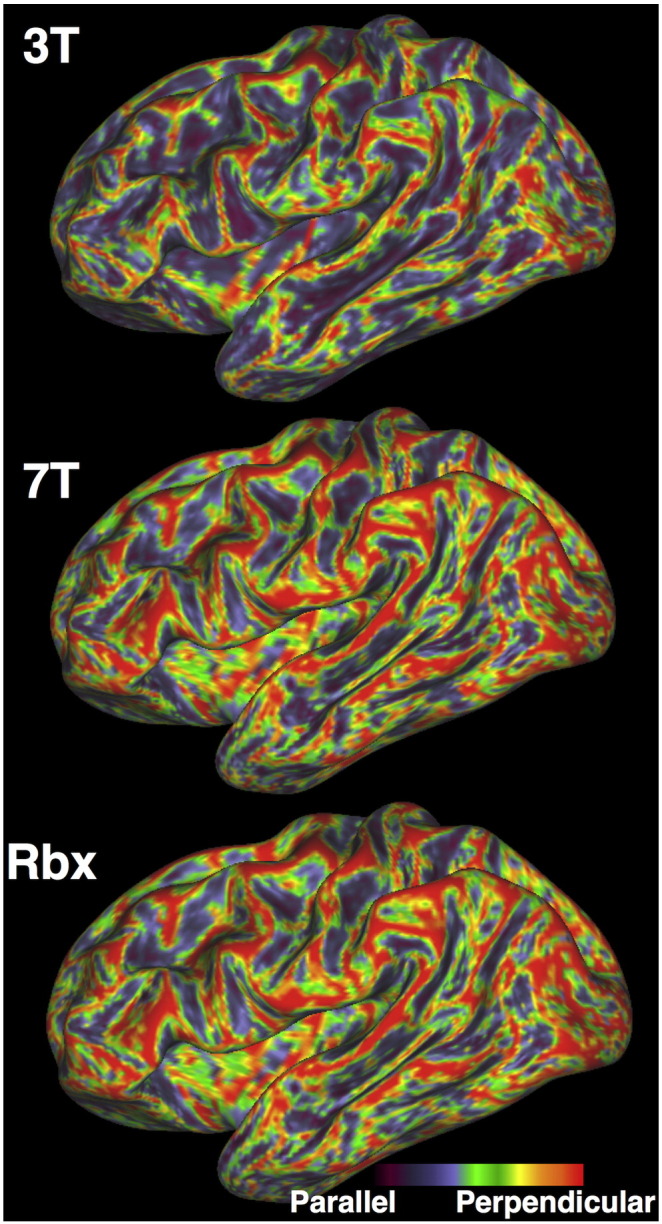
Angle between surface tangent and fibre orientations at the inflated WM/GM boundary surface. Orientations have been estimated as before using voxel-wise deconvolution on the 3T alone, voxel-wise deconvolution on the 7T alone, RubiX deconvolution on both 3T and 7T. For every surface vertex, the maximum dot product between fibre orientations (with volume fraction f > 5%) at this location (within a voxel size distance from the vertex location and on either side of the surface) and the surface normal is computed. This is then converted to the colour-code angle shown on the inflated surface. To aid visualisation of these qualitative illustrations, Gaussian smoothing on the surface with 1 mm FWHM was performed for all cases. (HCP Subject ID: 158035).

**Fig. 8:**
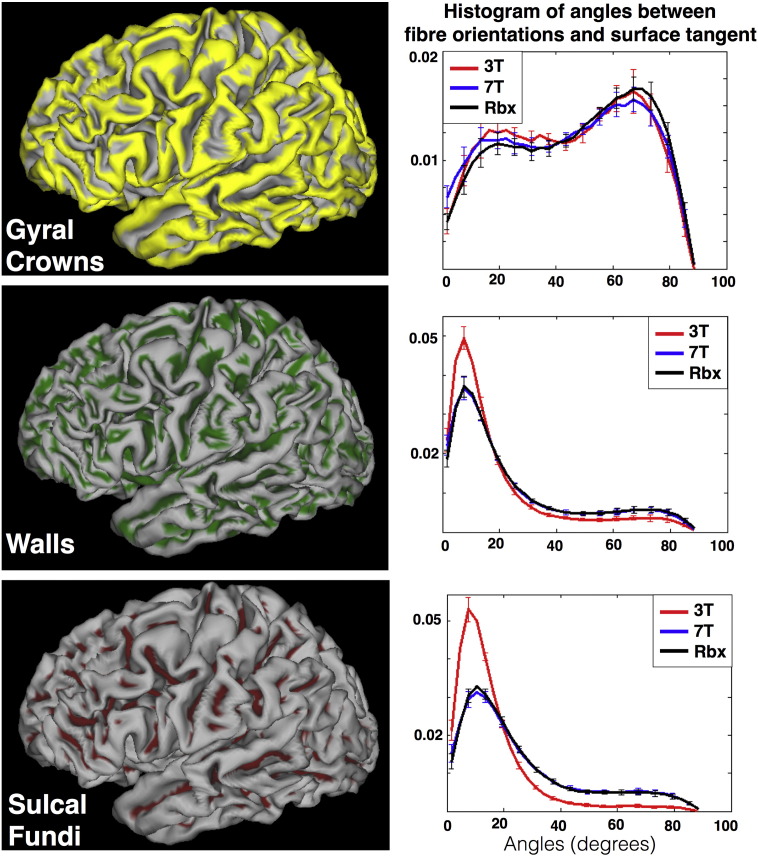
Normalised histograms of angles between fibre orientations and surface tangent at three subregions of the WM/GM boundary surface: gyral crows (top), gyral walls (middle) and sulcal fundi (bottom). Orientations have been estimated as before using voxel-wise deconvolution on the 3T alone (red), voxel-wise deconvolution on the 7T alone (blue), RubiX deconvolution on both 3T and 7T (black). For every surface vertex, the maximum dot product between fibreorientations (with volume fraction f > 5%) at this location and the surface normal is computed. This is then converted to the angles shown. For every bin of the histograms, the mean angle across ten subjects is shown. The error bars indicate the standard deviation across these subjects.

As shown qualitatively in Fig. 2, the higher resolution 7T data support clearer fibre spreading patterns towards the cortex at the transition between WM and GM. Fig. 7, Fig. 8 clarify this difference further and illustrate that this feature is retained after data fusion. This is desirable behavior as the axonal patterns at the boundary between white and grey matter indeed support such spreading geometries (Miller et al., 2011, Heidemann et al., 2012, Van Essen et al., 2013a), which are often missed by conventional, lower resolution dMRI (Sotiropoulos et al., 2013a, Reveley et al., 2015).

More specifically, Fig. 7 presents the angle between the estimated fibre orientations and the tangent plane to different locations of the WM/GM boundary surface. For each surface vertex, the estimated fibre compartments from the closest voxel were found and the compartments that had a volume fraction larger than 5% were retained. The orientation of the compartment most parallel to the surface normal was used to derive the presented angle. As shown in Fig. 7, the orientations estimated from the 3T (top) are mostly parallel to the surface and only perpendicular to it at the top of the gyral crowns. The orientations estimated from the 7T (middle) are more often close to perpendicular to the surface and are in general less parallel to the surface. This follows more closely the spreading fibre patterns to the cortex suggested by histology (Van Essen et al., 2013a) and allows in principle more connections to reach the cortex at places other than the gyral crowns. The orientations estimated from RubiX (bottom) follow a similar behavior to the 7T.

To further characterise these differences we divided the WM/GM boundary surface into three subregions. We used the mean curvature at every vertex to differentiate between gyral crowns (high positive curvature), gyral walls (roughly zero curvature) and sulcal fundi (high negative curvature). We then obtained for each subregion a histogram of the angles subtended between the estimated fibre orientations and the surface tangent (Fig. 8). The difference between the 3T and 7T data is minimal at the gyral crowns, but it is considerable at the gyral walls and sulcal fundi. The probabilities of angles smaller than 20° are respectively 17% and 40% higher with the 3T. The probabilities of angles higher than 60° are respectively 40% and 150% higher with the 7T. The histograms for the RubiX estimates follow very closely the 7T ones.

**Fig. 9:**
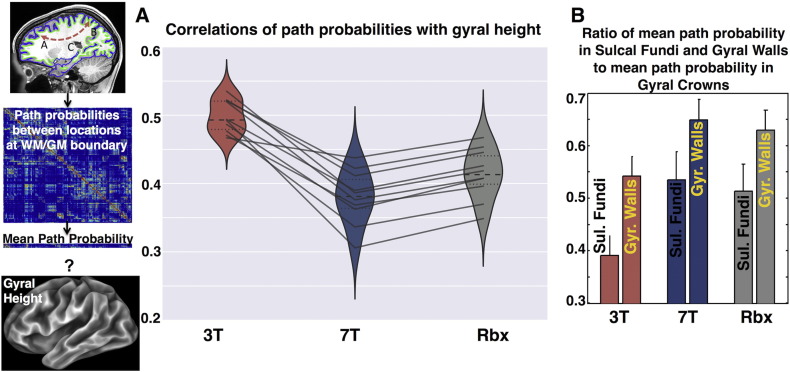
A) Correlations of mean path probabilities on the WM/GM boundary surface with gyral height (“sulc” map obtained from FreeSurfer). Violin plots illustrate the distribution of these correlations across ten subjects and for three different methods (3T, 7T, Rbx). The dashed lines within the plots show the median of the distribution and the dotted lines the inter-quartile range. The individual points are also shown and the solid lines connect the points corresponding to the same subject, across the three different methods. The mean path probabilities were estimated using tractography. As shown on the left column, probabilistic tractography was seeded at every brain tissue voxel C (~ 190,000 seeds, 3000 samples per seed, curvature threshold = 80°, Pial surface termination mask, WM/GM boundary surface allowed to be crossed only twice). Path probabilities were then recorded between pairs of 60,000 vertices on the WM/GM boundary surface to give a comprehensive connectome matrix. The mean path probability of every vertex was obtained by taking the average across the rows of the connectome. The logarithm of the path probabilities was correlated with the gyral height at the respective vertex locations. Gyral height is high for gyral crowns and low for sulcal fundi. B) Mean path probabilities at different regions of the WM/GM boundary surface (sulcal fundi, gyral walls), normalised to the corresponding mean path probability at the gyral crowns. The mean path probability values obtained on the WM/GM surface as in (A) were grouped into the three subregions described in Fig. 8. The average across these subregions was obtained and the ratio of these values at the sulcal fundi and the gyral walls to the value at the gyral crowns is presented. The bars show the mean and standard deviation of the ratios across ten subjects.

We then checked how these differences affect the behavior of tractography towards the cortex. In particular we hypothesized that the gyral bias, the anatomically unjustified (at least up to a certain extent) tendency of streamlines to preferably terminate at the gyral crowns (Van Essen et al., 2013a), is reduced with the 7T/RubiX orientations. We estimated a connectome for each subject, by seeding everywhere in the brain and recording the path probabilities between pairs of locations on the WM/GM boundary surface. We then obtained for every surface vertex a mean path probability from that vertex to any other vertex. This probability is representative of how “visited” each vertex is; in the presence of a strong gyral bias, gyral crowns will be more visited than other areas of the cortical ribbon. Subsequently, we examined how these probability values correlate with a probe of cortical geometry/folding. We chose as a probe the “gyral height” (“sulc” map as obtained from the HCP pipelines (Glasser et al., 2013) and FreeSurfer (Fischl, 2012)); this is the normalised signed linear distance of a vertex from the mid-surface between gyri and sulci (positive for gyral crowns, roughly zero for gyral walls and negative for sulcal fundi). Fig. 9A shows violin plots of the correlation coefficients between the logarithm of the mean path probability and the sulc values. Using the 3T data leads to higher correlations of the streamline termination points with cortical folding, meaning that more streamlines are concentrated at locations with high sulc values, i.e. at the gyral crowns. The use of 7T data brings down this correlation, allowing a more even distribution of the streamline terminations throughout the surface. Data fusion preserves this trend (~ 8% increase in the median correlation compared to the 7T, but still ~ 20% decrease compared to the 3T).

Fig. 9B quantifies the reduction of the gyral bias in a different way. The WM/GM surface was divided into the three subregions, shown in Fig. 8 (gyral crowns, gyral walls, sulcal fundi). The average path probability within each of these regions was obtained and the ratio of the values at the sulcal fundi and the gyral walls to the value at the gyral crowns is shown for each method and across 10 subjects. A 37% (20%) increase of the connections ending up at the sulcal fundi (gyral walls) relative to the connections ending up at the gyral crowns is obtained with the 7T when compared to the 3T results. Very similar behavior is retained with RubiX.

In summary, the results suggest that the 3T & 7T data fusion brings sensitivity for detecting crossings to levels close to the ones observed in the 3T data. At the same time the fusion matches desirable features of the 7T data, such as reduced gyral bias.

### Tractography examples

We next compared the effect of data fusion on tractography results. Given the differences in spatial resolution between the methods explored, we defined seed (as well as constraint) masks in the standard MNI 1 mm space. We also binned the tractography-estimated path distributions in the same space. Transformations between the MNI and the native spaces were obtained as in Glasser et al. (2013) using a non-linear registration of the T1-weighted images acquired at 3T.

**Fig. 10:**
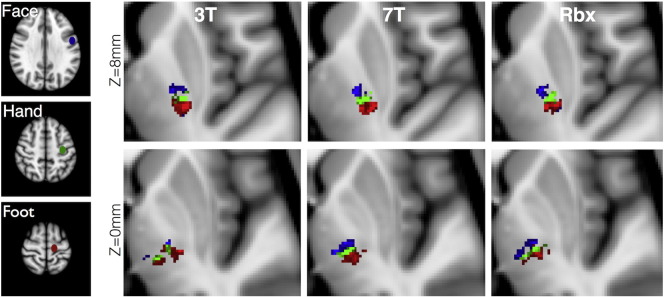
Somatotopy of motor projections in the internal capsule and the thalamus. Tractography was seeded from the foot, hand and face area of the primary motor cortex, as shown on the left. Axial views of the path distributions are shown for the different methods at two different Z positions. All results are shown in the standard MNI 1 mm space to allow direct comparisons. Path distributions for the foot and face area have been thresholded at 1% (3% threshold has been used for the hand area).

Fig. 10 illustrates differences between the three different approaches (3T alone, 7T alone, RubiX 3T & 7T) when mapping different parts of the motor projections using probabilistic tractography. Seed regions were defined at the foot, hand and face area of the motor cortex. Paths going through the posterior limb of the internal capsule and the thalamus were retained. An exclusion mask covering the midsagittal plane was also used. A fine somatotopy is expected in the projections of the motor paths both in the internal capsule (Fig. 10, top row) and in the thalamus (Fig. 10, bottom row) and this was revealed with all methods. Projections from the face area in the thalamus were not clearly identified with the 3T data and were mixed with paths from the foot area. The separation was clearer using 7T and was retained after data fusion.

**Fig. 11:**
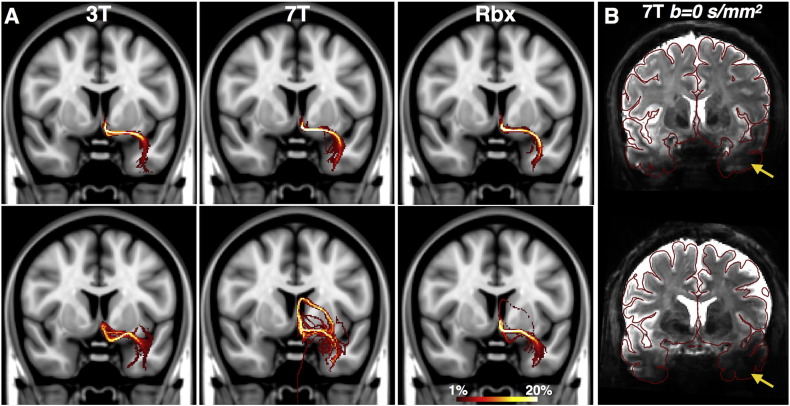
A) Maximum intensity projections along the coronal plane of the distribution of the limbic paths connecting subgenual white matter to the amygdala. Each column corresponds to probabilistic tractography results using fibre orientations estimated from the three different methods (voxelwise 3T deconvolution, voxelwise 7T deconvolution and RubiX deconvolution). The two rows correspond to different subjects (HCP Subject ID: 158035 — top, 109123 — bottom). B) Mean *b* = 0 image of the 7T data for the two subjects presented in (A) (HCP Subject ID: 158035 — top, 109123 — bottom). The red outlines represent the pial surface. Subjects with larger brain sizes have larger B1 inhomogeneity artifacts. These lead to more severe signal dropouts in the temporal lobes (yellow arrows).

Fig. 11A illustrates the ability to resolve a thin tract, the limbic pathways of the subgenual white matter to the amygdala (Mayberg et al., 2005). All methods perform a reasonable estimation as shown for an exemplar subject (top row). However, for some subjects (particularly with larger than average brain sizes) the 7T data support much noisier projections to the amygdala than the 3T (bottom row). Larger brain sizes lead to worse SNR (also larger heads require higher voltages to achieve the desired flip angles, which cannot be reached due to SAR constraints), which amplifies the signal dropouts in the inferior temporal regions due to B1 inhomogeneities (Fig. 11B, also artifacts shown in Fig. 2). The RubiX model seems beneficial in resolving such a situation, as the 3T data assist orientation estimation in the absence of usable 7T data.

**Fig. 12:**
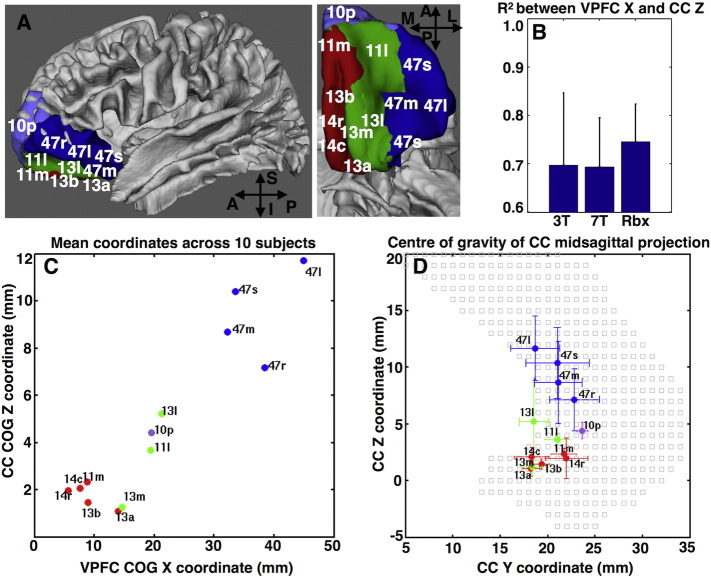
Replication of the organisation pattern of prefrontal cortex commissural projections using HCP data. A) The ventral prefrontal cortex (vPFC) has been divided into 13 subregions according to Ongur et al. (2003). The regions are shown from a sagittal perspective, as well as from below (A: anterior, P: posterior, S: superior, I: inferior, M: medial, L: lateral). The organisation pattern predicts that the more lateral the position of a subregion is, the more superior in the corpus callosum (CC) its commissural projection will be. Tracers injected in the vPFC, as well as macaque post-mortem tractography follow this pattern with a very strong correlation between the X position of the centre of gravity (COG) in the vPFC and the Z position of the COG of the callosal projection (Jbabdi et al., 2013). B) The coefficient of determination R^2^ between the vPFC X and the midsaggital CC Z position, when performing tractography using the fibre orientations estimated from the three different approaches. Mean and standard deviation shown across ten subjects. For each subject, the COG is computed for each vPFC mask (since these are binary masks, the COG is simply the mean of the coordinates of all voxels contained in the mask). Also, for each mask the respective callosal path distribution is obtained using tractography. The COG of the distribution is obtained on the midsagittal plane (since this is a path distribution, the COG is the centre of mass of this distribution, i.e. coordinates are weighted by the respective probabilities). The squared correlation coefficient between the X coordinate of vPFC COG and the Z coordinate of the respective CC COG is then computed. The barplots show these values across ten subjects. RubiX results have on average the highest and less variable R^2^ compared to the other two methods. C) Mean coordinates across ten subjects of the COG of the midsaggital CC projections from each vPFC subregion when using RubiX. D) Mean and standard deviation across subjects of coordinates of the midsaggital CC projections' COG when using RubiX. The grey squares delineate the anterior part of the corpus callosum. Coordinates are all reported in MNI space. For all tractography a waypoint was used for the anterior CC. For each vPFC seed, the rest subregions were used as exclusion masks.

We finally checked how well we could replicate an organisational rule learned from tracers using the orientation estimates obtained from the different methods. Injection of retrograde tracers in the ventral prefrontal cortex (vPFC) of the macaque reveals a certain pattern for the commissural projections. The more lateral the injection site is at the cortex, the more superior the commissural projection ends up within the corpus callosum (R^2^ > 0.9 between the medial-lateral vPFC position and the midsagittal inferior–superior position in the corpus callosum). This pattern has been augmented and validated with tractography in the post-mortem macaque brain (Jbabdi et al., 2013). Fig. 12 shows that we can replicate such a pattern with the HCP data (the results with RubiX are shown in the figure, but a similar trend is revealed for the other two methods). Across ten subjects we computed the correlation coefficient of vPFC X-position and CC Z-position. We obtained a higher and less variable R^2^ (mean R^2^ = 0.697, 0.693 and 0.746 for 3T, 7T and RubiX respectively) when using the data fusion model.

## Discussion

We presented a framework for combining diffusion MRI data acquired using different k-space and q-space sampling protocols. We explored complementary features of the available HCP data acquired using different scanners and magnetic field strengths (3T and 7T) (Sotiropoulos et al., 2013c, Vu et al., 2015). We then used a data fusion approach to estimate fibre orientations in a way that preserved desired features from the combined datasets. Despite the fact that individual HCP data are of very high quality on their own, we showed benefits when performing a joint analysis, which reflect the better angular contrast and angular resolution of the 3T and the better spatial resolution of the 7T.

The RubiX generative model used for this data fusion has been presented before for combining dMRI acquired using the same 3T scanner, at the same b-value, but different spatial resolutions (Sotiropoulos et al., 2013b). Here, we modified the local and spatial representations (Eq. (5), (6)) to allow data, different in both q- and k-space sampling, to be combined. This tackles a complex trade-off in diffusion MRI acquisitions, between SNR, spatial resolution and angular contrast. We illustrated the benefits in the context of the HCP and of combining data from different scanners, but data from the same scanner using different sampling approaches could also be combined, as originally shown in Sotiropoulos et al. (2013b).

Other groups have also recently considered the advantages of data fusion within the same modality (dMRI). In Fan et al. (2015), similar to our study, the benefit of combining data with different sampling protocols is illustrated when focusing on tractography near the cortex. A relatively low-resolution & high b-value dataset (aimed at deep white matter) is combined with a high-resolution & low b-value dataset (aimed at regions near the cortex). However, their combination reduces down to a binary selection of data using the WM/GM interface as a decision boundary, which leads to half of the data being thrown away. Here we achieve a similar aim by using a generative model of both datasets that are combined without the need for performing such binary decisions.

In Alexander et al. (2014), the HCP data are used to learn a mapping of high-resolution spatial patterns to low-resolution representations. By detecting these patterns in unseen conventional dMRI the authors transfer features learned from the bespoke high-quality HCP images to standard low-resolution diffusion images and super-resolve them. This is effectively an advanced approach to data interpolation, which has been shown to improve the geometrical resolution content of dMRI (Dyrby et al., 2014). Even if an exhaustive comparison with interpolation methods is outside the scope of this study, we confirmed that certain exquisite features of the high-resolution data could not be obtained via simple interpolation (see Supplementary Material, Figure S6). Therefore, our data fusion offers more than interpolation and, similar in spirit to Alexander et al. (2014), complements image quality between the 3T and 7T datasets.

Using the RubiX framework, we also extend the idea of classical voxel-wise spherical deconvolution (Anderson, 2005, Behrens et al., 2007, Dell'Acqua et al., 2007, Tournier et al., 2007). Benefits from using spatial information in the deconvolution have been illustrated, either as a means to regularise the estimates (e.g. (Goh et al., 2009)) or as a way to infer within-voxel asymmetry (Reisert et al., 2012). By coupling a spatial and a local model, we also show that complementary datasets can be combined and this complementarity is reflected into the estimates. We also observe that more precise estimates are obtained using data fusion (Fig. 6). We need to point out that this feature is expected and not surprising in this application, as the fused data have larger total scan time than the individual datasets. But we have shown before the benefit of increased precision and accuracy in a comparison where fused data were matched for scanning time with the individual datasets (Sotiropoulos et al., 2013b).

### Model assumptions and simulations

The current model representations have certain assumptions. The local model (Eq. 5) is aimed to deconvolve the fODF using a parametric approach. This captures isotropic partial volume and also permits the estimation of features of the convolution kernel. The anisotropy of an axially symmetric tensor response is estimated with a prior on that parameter learned from the data. However, we assume and fit a single average anisotropy of that convolution kernel across all *b*shells. In reality, the average anisotropy would increase and the ideal kernel will change with *b*value. Incorporating an extension similar to Jeurissen et al. (2014) would increase the accuracy. Nevertheless, we extend here the commonly used practice in deconvolution (Tournier et al., 2007, Jeurissen et al., 2014), by allowing some spatial variability of the convolution kernel with an informative prior instead of using a single kernel for all voxels.

The isotropic compartment used in the local model is a phenomenological representation, using a Gamma distribution of diffusivities, as in Jbabdi et al. (2012). It can capture the non-mono-exponential decay of the signal with *b* value. Therefore, it can accommodate the combination of data with multiple *b* values. Other models may have a more direct biophysical interpretation (for instance the bi-exponential model (Clark et al., 2002), kurtosis imaging (Jensen et al., 2005), the stretched exponential model (Bennett et al., 2003) or models that explicitly represent restriction (Stanisz et al., 1997, Assaf et al., 2008)), but the Gamma distribution representation is more compact adding only one extra parameter.

The spatial model (Eq. 6) assumes that T1 and T2 vary smoothly in the neighborhood of voxels whose signal is combined. Furthermore, relaxation, which can be different at different acquisition timings (TE, TR) and field strengths, is not represented explicitly in either the spatial or the local model. We used simulations to assess the impact of these simplifications (Supplementary Material, Figure S2). We refer the interested reader to the Supplementary Material for the full details and we present here the main findings. Simulations showed that the impact is minimal for estimated orientations, which are the parameters of interest for this framework. The most profound effects were observed at boundaries between tissue types (particularly at WM/CSF borders), on the estimated diffusivities and volume fractions. Given that we use shrinkage priors on the volume fractions, such deviations are less likely to induce false positive crossings. A worst-case scenario is getting the fractions wrong when the true anisotropic volume fractions are small (in the order of ~ 5%), which can induce false negatives in the complexity of the fODFs. Yet, our estimates of complexity (Fig. 6), even at this high resolution, fall within the expected range as estimated from data of conventional spatial resolution (Jeurissen et al., 2013).

The spatial model can be improved in a number of ways. For instance, differences between the combined datasets in PSF blurring along the phase-encoding direction are currently ignored. These could be incorporated into the model. A preliminary attempt to incorporate the PSF into the model has been recently shown in (Pisharady et al., 2015). Furthermore, tissue maps (obtained e.g. from a structural segmentation) could be used for a more accurate partial volume representation that accounts for tissue-specific relaxation rates. Nevertheless, the simple representation used in this study predicts well measurements made at both field strengths and with different protocols (Fig. 3, Fig. 4).

An underlying assumption of the presented framework is that the fused datasets are well aligned. Of course, given the 4D nature of diffusion datasets, alignment of diffusion-weighted volumes in the presence of distortions and head motion is a prerequisite even for traditional voxel-wise analysis (Nilsson et al., 2015, Graham et al., 2016). In this study, we have used state-of-the-art methods to align the datasets, including distortion correction (Andersson et al., 2003, Andersson and Sotiropoulos, 2016), boundary-based registration (Greve and Fischl, 2009) to reduce sensitivity to residual distortions and unwarping of shape distortions caused by gradient non-linearities (Glasser et al., 2013). Simulations of relatively worst-case scenarios of residual misalignments (see Supplementary Material, Figure S3) confirmed that performance deteriorates with misalignment, but even for bad alignment scenarios the model does not behave in an unpredictable manner and performance is reasonable.

### Limitations and future work

A limitation in our implementation is the requirement of fusing data acquired with voxel sizes of one dataset being exact multiples of the sizes of the other. We have made this simplification to allow parallelisation in the inference and achieve computation within realistic time frames. This renders the HCP data (obtained from protocols aimed to push the limits as much as possible at every field strength within the allotted scanning time) slightly sub-optimal for our current implementation. Yet, in Sotiropoulos et al. (2013b) we evaluated the effect of such a setup (with LR voxel volume being twice the HR one). We found that the RubiX estimates have spatial specificity very close and representative of the HR grid they are obtained at, while the LR data constrain the solution space. The price for those LR constraints was found to be in the order of 5–10% resolution loss in the estimates, which we introduce in the case of HCP data when downsampling the 3T. We anticipate that removing these implementation-related requirements will further improve the spatial specificity of the estimates. Nevertheless, the angular contrast that the 3T data carry complemented with the higher spatial resolution of the 7T is beneficial in many instances compared to analyzing the original 3T data alone without downsampling (Fig. 7, Fig. 8, Fig. 9, Fig. 12).

The coupling of the datasets during estimation is achieved by the spatial model, but also by the use of orientation priors. The exact hyperparameters used in the Watson priors have an influence on the accuracy and precision of the estimates and the spatial regularisation induced (Sotiropoulos et al., 2013b). We have used the hyper-parameters suggested in Sotiropoulos et al., (2013b), as these values were also used to evaluate the induced spatial regularisation and resolution loss described above.

We should finally point out that although the HCP data obtained on the same subjects at 3T and 7T provide insights into the effect of the field strength on dMRI, these data should not be considered a direct field strength comparison. The 7T dMRI was allotted 40 min of total scan time, restricting it to 2 shells versus 3 shells and 55 min of scan time for 3T. The TR was also longer at 7T leading to fewer q-values per shell because of power deposition (SAR) constraints (which can be in principle overcome with more recent technical developments). Maximal gradient strengths available would have a major impact on such a comparison, with higher maximal gradients being much more important at the higher field strength. Rather, the present study informs more on the effects of data with higher spatial resolution versus data with higher b-values and more angular sampling and the benefits from their joint analysis. Future studies can improve on the already highly advanced HCP data by taking into account the results obtained in this study.

## Appendix A Supplementary data

Supplementary material. Image 1
