# Supplementary material for: Fusion in diffusion MRI for improved fibre orientation estimation: An application to the 3T and 7T data of the Human Connectome Project
Source: Neuroimage. 2016 Jul 1;134:396–409. doi: 10.1016/j.neuroimage.2016.04.014 (PMC6318224; doi:10.1016/j.neuroimage.2016.04.014)
Supplement: Supplementary file 1 — Supplementary material. [file mmc1.pdf]

Fusion in Diffusion MRI for Improved Fibre Orientation Estimation: An application to the 3T and 7T data of the Human Connectome Project

Sotiropoulos SN, Hernández-Fernández M, Vu AT, Andersson JL, Moeller S, Yacoub E, Lenglet C, Ugurbil K, Behrens TE, Jbabdi S

Supplementary Material

1. Simulations

A simulated phantom with crossing bundles was used to evaluate the performance of the data fusion model. Extensive simulations for assessing the behavior of the original RubiX model were presented previously in (Sotiropoulos et al., 2013). Here, we explore the robustness of the technique particularly at tissue interfaces, where differences in relaxation times T_1 and T_2, as well as echo time (TE) and repetition time (TR), with field strength can have the most profound effects.

We simulated two crossing white matter bundles embedded within isotropic background (Fig. S1). The bundles bordered areas that mimicked grey matter (GM) and cerebrospinal fluid (CSF). The phantom included two slices with the elevation angle of the orientations being 0 for the first and 10 for the second slice. That ensured that orientations within 3D voxel neighborhoods were not replicated and were changing in all dimensions. To consider effects of field strength, two versions of the phantom with strength-dependent parameters were produced (see Table 1).

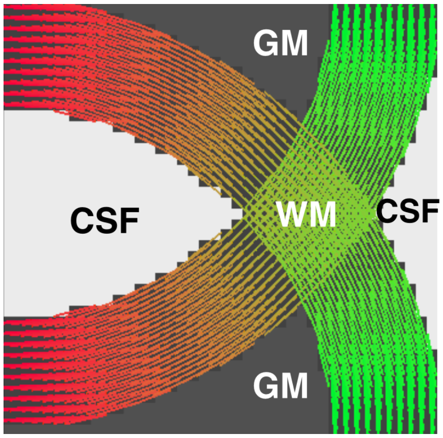

Figure S1. Phantom used for numerical simulations. Two crossing bundles were used to simulate white matter (ground-truth orientations are shown color-coded, red for left/right, green for up/down). These bordered with areas that simulated cerebrospinal fluid (CSF) and areas that simulated grey matter (GM).

First, the relaxation-free, noise-free signal at each WM voxel was simulated using a mixture of signals obtained from diffusion tensors. Specifically, in non-crossing regions

an isotropic tensor and an anisotropic axially symmetric tensor were used (with mean diffusivity d_{WM} for both and eigenvalues (λ_1, λ_2) for the latter as shown in Table 1). The volume fraction for the anisotropic tensor was f_{sum}=0.6. In crossing regions, two axially symmetric tensors were used with volume fractions 0.3 each, oriented along the crossing orientations. For the GM and CSF regions isotropic tensors were used (with mean diffusivities d_{GM} and d_{CSF} respectively). To mimic small differences in the diffusivities of each tissue type caused by the different acquisition protocols (differences in pulse duration δ, diffusion time Δ, maximum gradient strength, echo time, for instance (Qin et al., 2009)), we computed the DTI mean diffusivity of each tissue type, using the b=1000 s/mm^2 shell from the 3T and 7T in-vivo data and used these values in the simulations (see Table 1).

Relaxation effects were then added and the final signal S was:

$$S = S_{nr}\, e^{-\frac{TE}{T_2}}\left(1 - e^{-\frac{TR}{T_1}}\right),$$

with S_{nr} the simulated signal without any relaxation effects. TE and TR, as well as the b values and gradient directions used for the simulations were the ones of the actual 7T and 3T acquisitions (see Data Acquisition section). The relaxation times at different field strengths were obtained from the literature (Table 1). For the 7T, the T_2 for WM and GM were obtained from (Yacoub et al., 2003) and for CSF from (Wyss et al., 2013), while T_1 values were found in (Rooney et al., 2007). For the 3T, the T_2 for WM and GM were obtained from (Stanisz et al., 2005) and for CSF from (Neelavalli et al., 2009), while T_1 values were found in (Wansapura et al., 1999) for GM and WM and in (Lin et al., 2001) for CSF.

Table 1. Parameters used for the simulations

	**3T**	**7T**
$\mathbf{T_{1WM}}$ (ms)	832	1220
$\mathbf{T_{1GM}}$ (ms)	1331	2130
$\mathbf{T_{1CSF}}$ (ms)	4000	4420
$\mathbf{T_{2WM}}$ (ms)	70	45
$\mathbf{T_{2GM}}$ (ms)	85	55
$\mathbf{T_{2CSF}}$ (ms)	2200	946
**TE** (ms)	89	71
**TR** (ms)	5500	7000
d_{WM} (mm^2/s)*	0.69 x 10^{-3}	0.65 x 10^{-3}
λ_2/λ_1 †	0.13	0.13
d_{GM} (mm^2/s)*	0.82 x 10^{-3}	0.79 x 10^{-3}
d_{CSF} (mm^2/s)*	2.5 x 10^{-3}	2.5 x 10^{-3}
f_{sum}	0.6	0.6
b values (s/mm^2)	1000, 2000, 3000	1000, 2000
GRAPPA ***R***	1	3
N_{PE}	86‡	200
BW_{pixel} (Hz/pix)	1490	1388

*Obtained from real data as the mean diffusivities estimated from the DTI model in the corpus callosum, the cortex and the CSF in b=1000 s/mm^2 3T and 7T data (mean of 10 subjects).

† As calculated from real data, see Figure 1.

‡ Assuming the PE field of view of the 3T acquisition (180 mm) and voxel dimension of 2.1 mm.

Finally, to reflect the different spatial resolutions at each field strength (7T are high resolution (HR), 3T are low resolution (LR)), the version of the phantom corresponding to the 3T parameters was downsampled by a factor of two in each dimension. This represents a scenario of having a ratio of 2 between the voxel size at HR and LR, as was done with the real data. To simulate Rician noise, zero-mean Gaussian signal was added in quadrature. For a given signal to noise ratio (SNR) at HR, a scaled SNR level was used for the LR data. The scaling was found using the following formula for a 2D EPI sequence (Haacke et al., 1999):

$$SNR \propto FS \times V \times \sqrt{\frac{N_{PE}}{BW_{pixel}}} \times \sqrt{1/R}\ ,$$

with FS being the field strength, V the voxel volume, N_{PE} the number of phase encoding steps, BW_{Pixel} the bandwidth per pixel in the readout direction and R the in-plane parallel imaging acceleration factor. Using the parameter values given in Table 1 and $V_{LR}=8V_{HR}$, we obtained a scaling of $SNR_{LR}=3.7585\ SNR_{HR}$.

Effect of ignoring relaxation in the data fusion model

The RubiX model ignores tissue relaxation effects and their difference at different field strengths. Using the simulator we explored the influence of this simplification on the estimates. We compared the accuracy of RubiX estimates in two scenarios, one where relaxation is not considered for signal generation and another where relaxation effects are considered as described above (dark blue vs orange bar plots in Figure S2. A case without relaxation but higher SNR is also shown as a baseline (light blue bar plots). Ignore for now the green bar plots, as they are explained in the next section). As expected, the performance of the model is best (smallest errors) for the data that match the model assumptions best, i.e. the data simulated without any relaxation effects. However, in the more realistic scenario of considering relaxation, RubiX also performs very well. The orientation estimation works almost identically between the two cases (with the exception of a small error increase of ~7% for orientations at WM/CSF borders, Fig. S2A). The main performance penalties are observed in other parameters (the diffusivity, the anisotropic volume fractions and the baseline signal intensity, Fig. S2, panels C-E) and at tissue interfaces (mostly at WM/CSF borders). The relative errors increase by 12%, 15%, 78% at WM/GM interfaces and 28%, 25%, 113% at WM/CSF interfaces for the diffusivity, anisotropic volume fraction and S_0 intensity respectively. However, in all cases, the absolute errors remain within reasonable limits (smaller than 5-10%). Therefore, the simplification made in the RubiX model regarding relaxation effects seems to be a reasonable one, particularly if the estimation of orientations is of interest.

Which spatial model?

The spatial model used in RubiX is given by Eq. (6). This models the signal attenuation at the lower resolution as the attenuation of the sum of the signals at the high resolution voxels that intersect a low resolution voxel. Alternatively, another model uses a weighted sum of HR attenuations to represent the attenuation at the lower resolution (Sotiropoulos et al., 2013). We evaluated the robustness of these two alternatives, when

relaxation effects (and differences due to field strengths) are taken into account. Figure S2 orange and green bar plots represent errors using the two models. The performance of the two models is very similar within WM, but their difference is prominent at the WM/CSF interfaces, where the latter model is much more sensitive and prone to error (as before the orientation estimates seem to be less sensitive, with mean errors less than 10° even at the interfaces, but large errors can occur in the other parameters). We therefore used the spatial model of Eq. (6) in our data analysis.

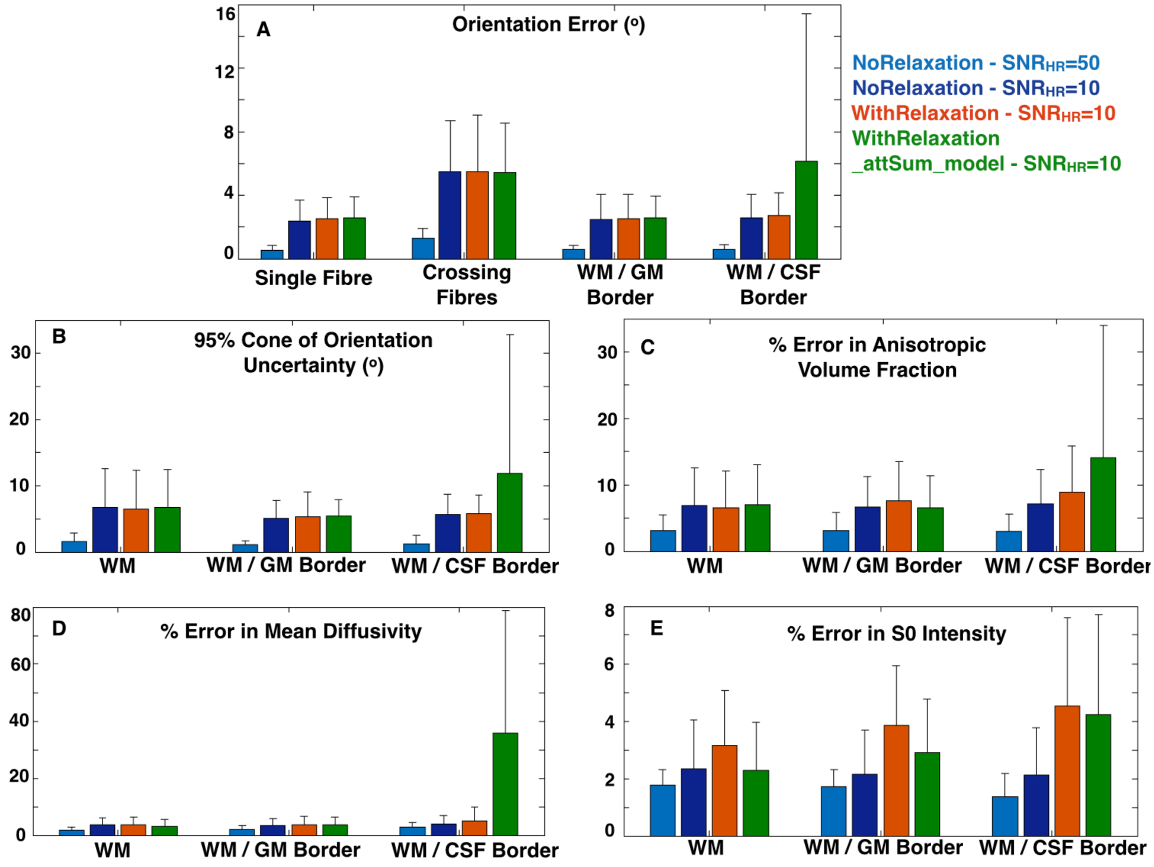

Figure S2. Errors in the estimates from the RubiX model on simulated data under different scenarios: when relaxation effects are ignored (dark blue), when relaxation effects are considered (orange), when relaxation effects are considered and an alternative spatial model is used (green). The data were simulated using SNR_{HR}=10, except a baseline case where relaxation effects are ignored and SNR_{HR}=50 (light blue). Mean and standard deviation of errors across indicated regions are shown in each case. A) Orientation error (in case of crossings, the mean error is shown). B) 95% cones of uncertainty for the estimated orientations (in case of crossings the mean is shown). C) Error in total anisotropic volume fraction, D) Error in Mean diffusivity. E) Errors in estimated baseline signal intensity S_0.

Effect of mis-registrations

The success of the data fusion relies on an accurate alignment of the datasets to be fused. Deviations from a good alignment can introduce errors to the results, due to

mixing of signals that correspond to different tissue locations; as they would do for traditional voxel-wise analysis, where diffusion-weighted volumes need to be aligned in the presence of distortions and head motion. We assessed the sensitivity of our framework to misalignments of the LR and HR datasets. With our current tools used for distortion correction and alignment (Glasser et al., 2013, Andersson and Sotiropoulos, 2016), misalignments in the order of 0.25 voxels are expected as a worst-case scenario (see Fig. 4 in (Graham et al., 2016)). We simulated two scenarios with misalignments along different directions (Figure S3). The misalignments were in the order of 0.25 LR voxels (or equivalently 0.5 HR voxels). Compared to a perfect alignment, the biggest absolute errors occur for the volume fractions and the diffusivities at the tissue interfaces. Orientation errors also increase, but even for the worst misalignment (and at SNR=10), the mean error remains less than 7°.

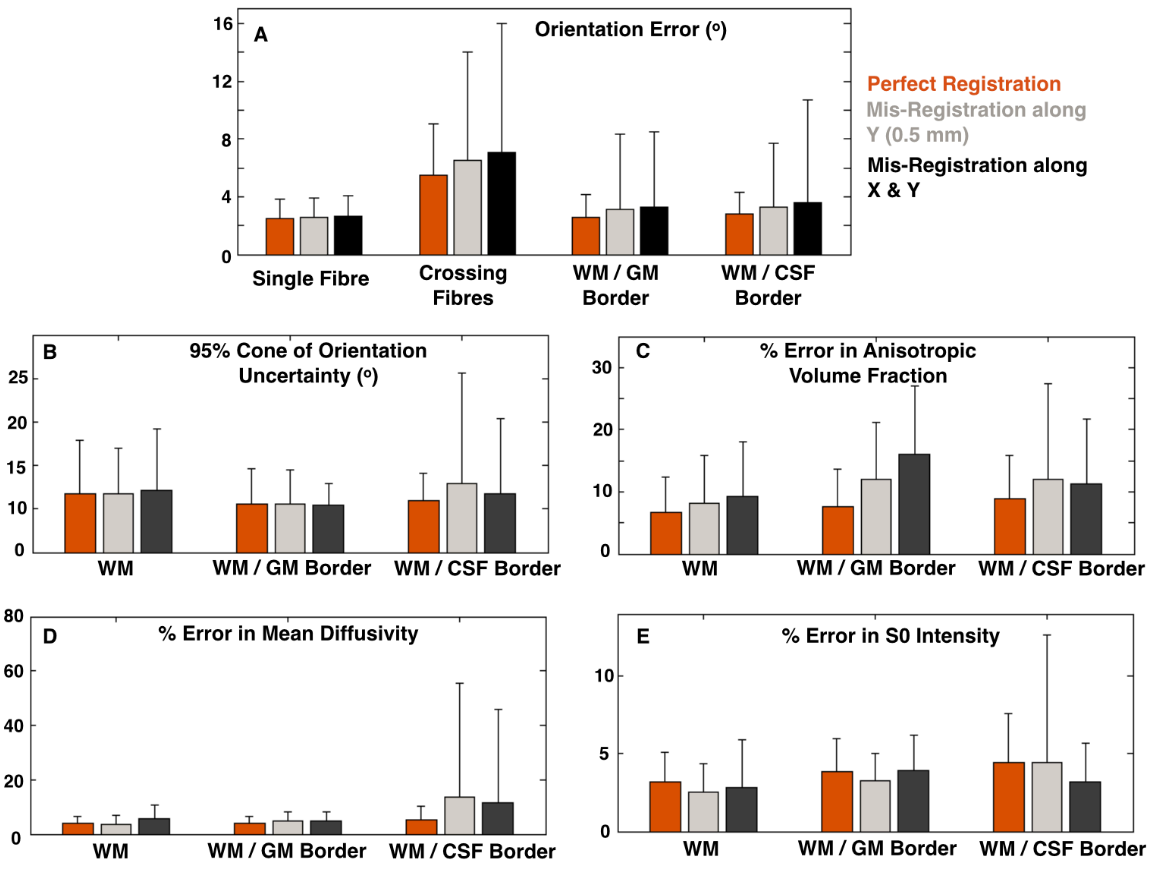

Figure S3. Errors in the estimates from the RubiX model on simulated data under different scenarios: when the LR and HR data are perfectly registered (orange), when the HR data are displaced half a voxel size along direction Y (grey) and when the HR data are displaced half a voxel size diagonally (along directions X & Y) (dark grey). Relaxation effects were considered. The data were simulated using SNR_{HR}=10. Mean and standard deviation of errors across indicated regions are shown in each case. A) Orientation error (in case of crossings, the mean error is shown). B) 95% cones of uncertainty for the estimated orientations (in case of crossings the mean is shown). C) Error in total anisotropic volume fraction, D) Error in Mean diffusivity. E) Errors in estimated baseline signal intensity S_0.

Simulations Summary

The main findings from our simulations are:

a) Ignoring relaxation effects and acquisition timings in the generative model is a simplification. However, this simplification influences more profoundly the estimates of diffusivity and volume fractions at the WM-CSF interface (Figure S2). Fibre orientations estimates are considerably less affected.

b) The spatial model proposed here (Eq. 6) behaves well at tissue interfaces and across different acquisition protocols (Figure S2), where integration of signals with potentially large differences may occur.

c) The proposed framework relies on a very good alignment of the fused datasets. But even when this is not the case, the model still performs reasonably well (Figure S3).

2. In-vivo Results

Similar to Figure 4 in the main paper, we present data and model predictions for inferior axial locations (where artefacts and inhomogeneities are worse, see frontal regions in Figure S4) and for a different subject with a larger brain size (which again inherently increases inhomogeneities particularly at high field, see Figure S5 and Figure 11). Even if the difference between model predictions and data is slightly higher in regions with more inhomogeneities, the trend is always the same with the model providing good predictions of both HR and LR datasets at all *b* values.

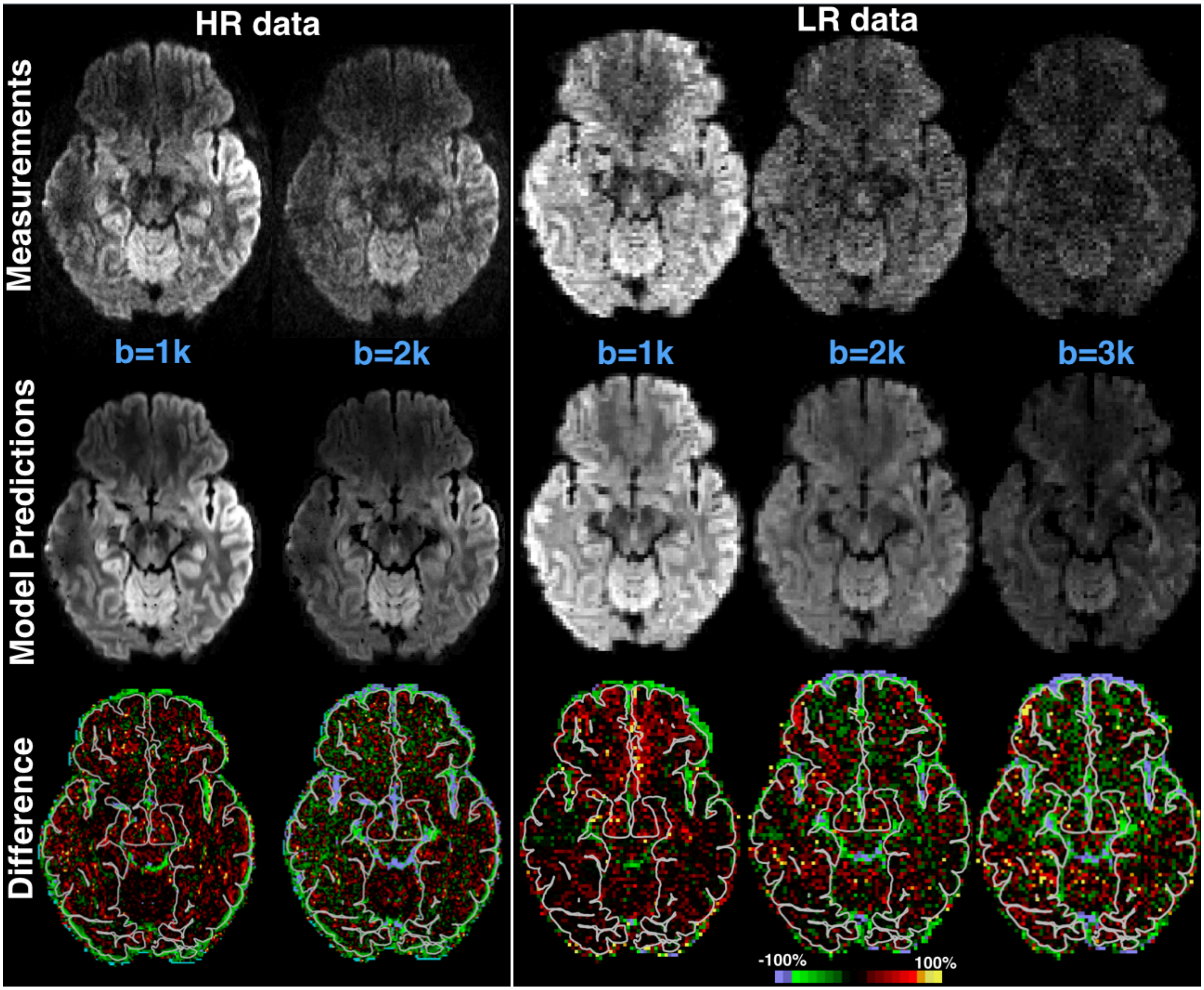

Figure S4. RubiX model predictions of different measurement volumes in the HR and LR data. Top row shows the actual measurements (the 3T data have been downsampled, as used in the estimation). Middle row shows the predictions. To obtain model predictions, the mode of the estimated posterior distribution was used for each model parameter. Bottom row shows the difference of the prediction minus the measurement expressed as a percentage of the measurement. The pial surface is shown as a gray line. (HCP Subject ID: 102311).

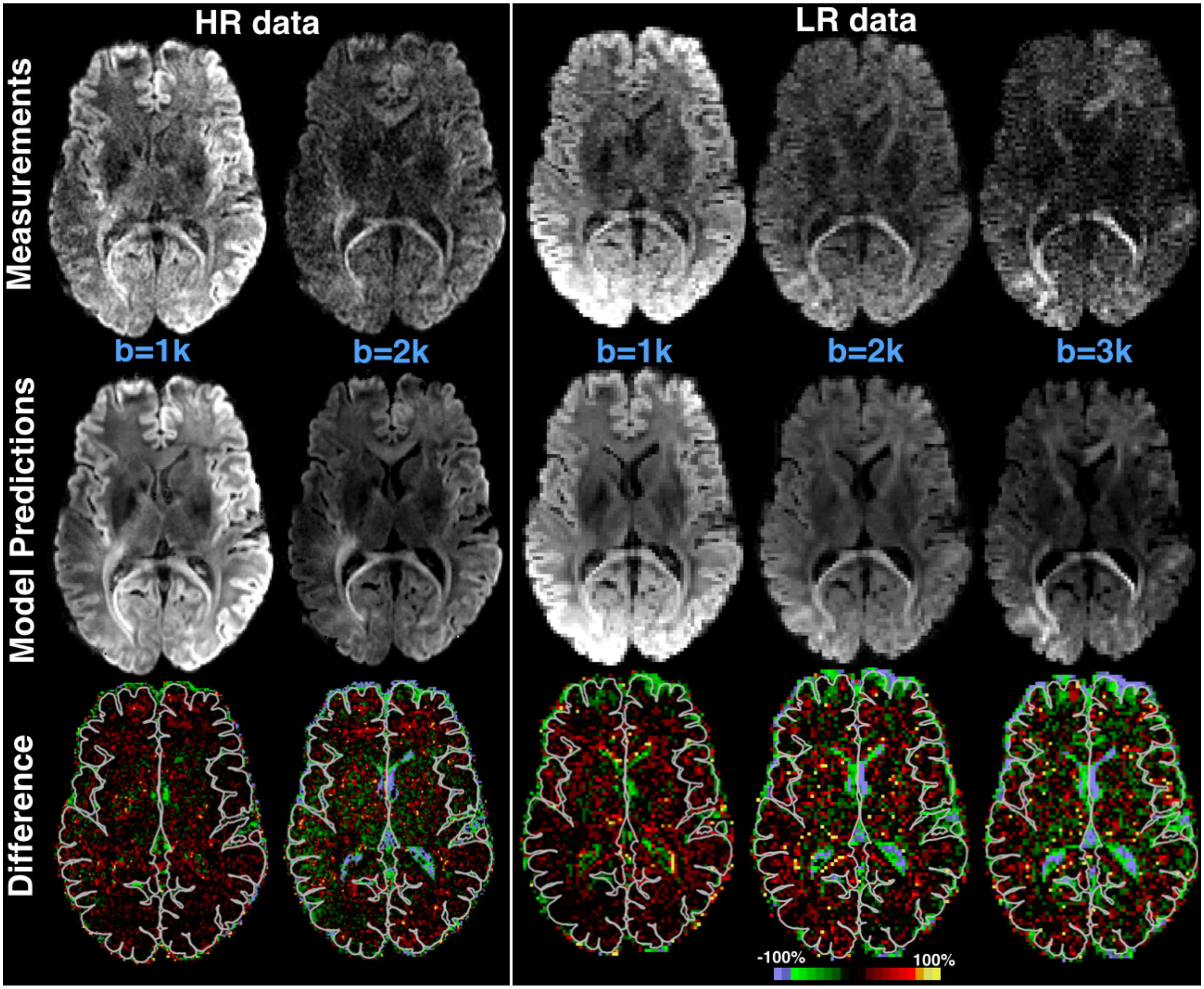

Figure S5. RubiX model predictions of different measurement volumes in the HR and LR data. Top row shows the actual measurements (the 3T data have been downsampled, as used in the estimation). Middle row shows the predictions. To obtain model predictions, the mode of the estimated posterior distribution was used for each model parameter. Bottom row shows the difference of the prediction minus the measurement expressed as a percentage of the measurement. The pial surface is shown as a gray line. (HCP Subject ID: 109123, larger brain size than 102311 and therefore inherently larger inhomogeneities at high field).

Supplementing Figure 7 in the main paper, we provide Figure S6 to show that certain exquisite features in the high-resolution data cannot be recovered by simple interpolation. We interpolated the 3T preprocessed data to the 7T spatial resolution (i.e. 1.05mm isotropic) using spline interpolation. We then estimated fibre orientations on the interpolated data using the voxelwise model and assessed how perpendicular or parallel to the white matter/gray matter boundary surface these orientations were (3T_spline). For reference we include the maps for all the other cases shown in Figure 7. As shown, even if the spline interpolation changes slightly the amount of orientations being perpendicular to the surface (e.g. area highlighted by the white arrow), it does not recover the features seen in the 7T data (noticeably more orientations perpendicular to the surface), which are preserved with RubiX data fusion.

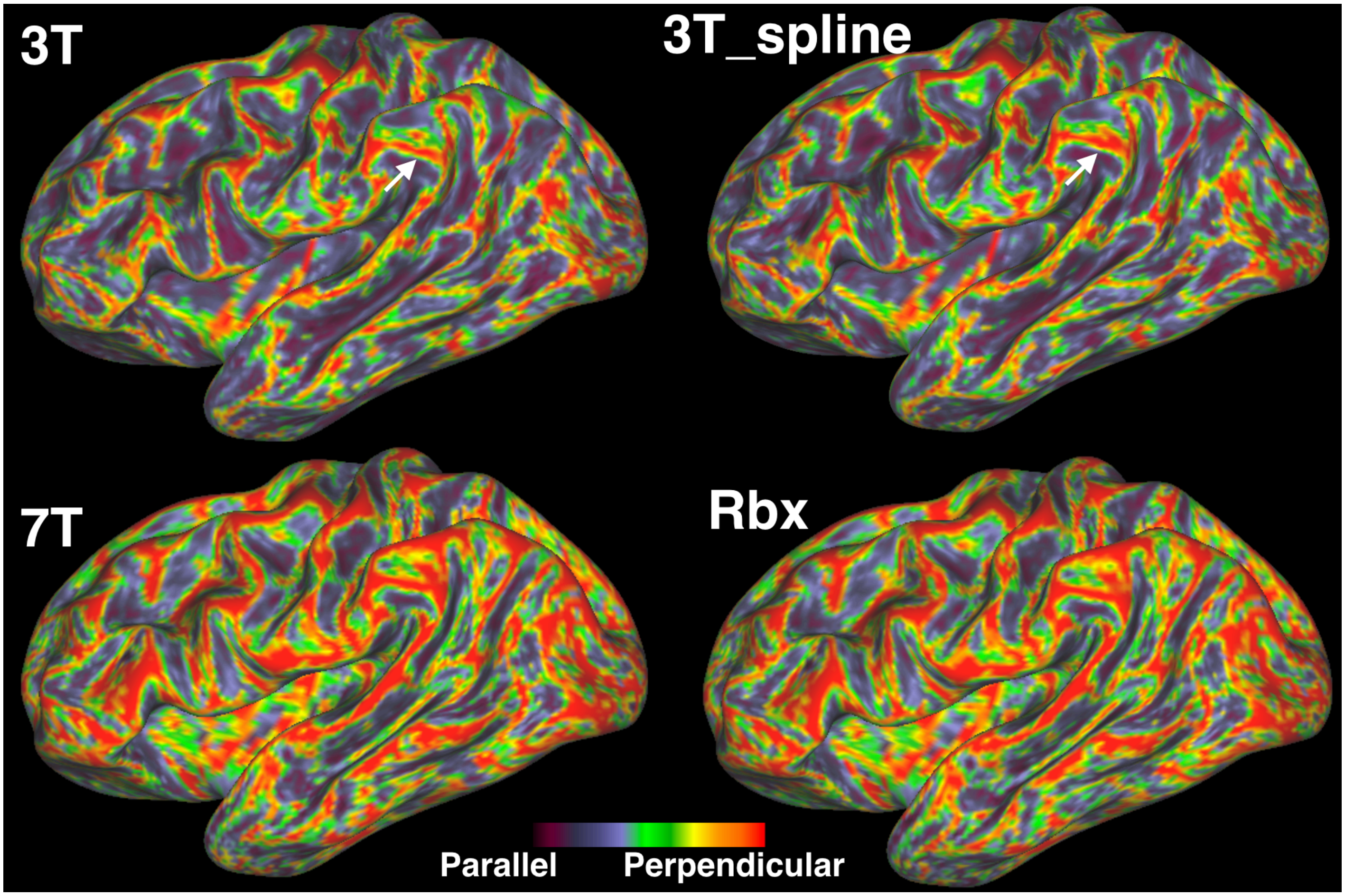

Figure S6. Angle between surface tangent and fibre orientations at the inflated WM/GM boundary surface. Orientations have been estimated as before using voxel-wise deconvolution on the 3T alone (estimates at 1.25mm isotropic resolution), voxel-wise deconvolution on a spline-interpolated version of the 3T data to the 7T resolution (estimates at 1.05mm isotropic resolution), voxel-wise deconvolution on the 7T alone (estimates at 1.05mm isotropic resolution), RubiX deconvolution on both 3T and 7T (estimates at 1.05mm isotropic resolution). For every surface vertex, the maximum dot product between fibre orientations (with volume fraction f>5%) at this location and the surface normal is computed. This is then converted to the colour-code angle shown on the inflated surface. To aid visualisation of these qualitative illustrations, Gaussian smoothing on the surface with 1mm FWHM was performed for all cases. (HCP Subject ID: 158035)

References

Andersson JL, Sotiropoulos SN (2016) An integrated approach to correction for off-resonance effects and subject movement in diffusion MR imaging. NeuroImage 125:1063-1078.

Glasser MF, Sotiropoulos SN, Wilson JA, Coalson TS, Fischl B, Andersson JL, Xu J, Jbabdi S, Webster M, Polimeni JR, Van Essen DC, Jenkinson M, Consortium WU-MH (2013) The minimal preprocessing pipelines for the Human Connectome Project. NeuroImage 80:105-124.

Graham MS, Drobnjak I, Zhang H (2016) Realistic simulation of artefacts in diffusion MRI for validating post-processing correction techniques. NeuroImage 125:1079-1094.

Haacke EM, Brown RW, Thompson MR, Venkatesan R (1999) Magnetic Resonance Imaging: Physical Principles and Sequence Design: John Wiley & Sons.

Lin C, Bernstein M, Huston J, Fain S (2001) Measurements of T1 Relaxation times at 3.0T: Implications for clinical MRA. ISMRM Proceedings 1391.

Neelavalli J, Haacke EM, Young IR, Bydder GM (2009) Contrast development and manipulation in MR imaging. In: Magnetic Resonance Imaging of the Brain and Spine - 4th Edition, vol. 1 (Atlas, S. W., ed), pp 25-47: Lippincott Williams & Wilkins, Wolters Kluwer.

Qin W, Yu CS, Zhang F, Du XY, Jiang H, Yan YX, Li KC (2009) Effects of echo time on diffusion quantification of brain white matter at 1.5 T and 3.0 T. Magn Reson Med 61:755-760.

Rooney WD, Johnson G, Li X, Cohen ER, Kim SG, Ugurbil K, Springer CS, Jr. (2007) Magnetic field and tissue dependencies of human brain longitudinal 1H2O relaxation in vivo. Magn Reson Med 57:308-318.

Sotiropoulos SN, Jbabdi S, Andersson J, Woolrich MW, Ugurbil K, Behrens TE (2013) RubiX: Combining Spatial Resolutions for Bayesian Inference of Crossing Fibres in Diffusion MRI. IEEE transactions on medical imaging 32:969-982.

Stanisz GJ, Odrobina EE, Pun J, Escaravage M, Graham SJ, Bronskill MJ, Henkelman RM (2005) T1, T2 relaxation and magnetization transfer in tissue at 3T. Magn Reson Med 54:507-512.

Wansapura JP, Holland SK, Dunn RS, Ball WS, Jr. (1999) NMR relaxation times in the human brain at 3.0 tesla. Journal of magnetic resonance imaging : JMRI 9:531-538.

Wyss M, Kirchner T, Ringenbach A, Prussmann K, Henning A (2013) Relaxation Parameter Mapping Adapted for 7T and Validation against Optimized Single Voxel MRS. ISMRM Proceedings 2464.

Yacoub E, Duong TQ, Van De Moortele PF, Lindquist M, Adriany G, Kim SG, Ugurbil K, Hu X (2003) Spin-echo fMRI in humans using high spatial resolutions and high magnetic fields. Magn Reson Med 49:655-664.
